# Synergistic Interactions of Cannabidiol with Chemotherapeutic Drugs in MCF7 Cells: Mode of Interaction and Proteomics Analysis of Mechanisms

**DOI:** 10.3390/ijms221810103

**Published:** 2021-09-18

**Authors:** Muhammad A. Alsherbiny, Deep J. Bhuyan, Mitchell N. Low, Dennis Chang, Chun Guang Li

**Affiliations:** 1 NICM Health Research Institute, Western Sydney University, Penrith, NSW 2747, Australia; D.Bhuyan@westernsydney.edu.au (D.J.B.); mitchell.low@westernsydney.edu.au (M.N.L.); D.chang@westernsydney.edu.au (D.C.); 2 Department of Pharmacognosy, Faculty of Pharmacy, Cairo University, Cairo 11562, Egypt

**Keywords:** cannabidiol, CBD, doxorubicin, docetaxel, paclitaxel, SN−38, vinorelbine, breast cancer, synergy, apoptosis, proteomics

## Abstract

Cannabidiol (CBD), a nonpsychoactive phytocannabinoid, has recently emerged as a potential cytotoxic agent in addition to its ameliorative activity in chemotherapy-associated side effects. In this work, the potential interactions of CBD with docetaxel (DOC), doxorubicin (DOX), paclitaxel (PTX), vinorelbine (VIN), and 7-ethyl-10-hydroxycamptothecin (SN−38) were explored in MCF7 breast adenocarcinoma cells using different synergy quantification models. The apoptotic profiles of MCF7 cells after the treatments were assessed via flow cytometry. The molecular mechanisms of CBD and the most promising combinations were investigated via label-free quantification proteomics. A strong synergy was observed across all synergy models at different molar ratios of CBD in combination with SN−38 and VIN. Intriguingly, synergy was observed for CBD with all chemotherapeutic drugs at a molar ratio of 636:1 in almost all synergy models. However, discording synergy trends warranted the validation of the selected combinations against different models. Enhanced apoptosis was observed for all synergistic CBD combinations compared to monotherapies or negative controls. A shotgun proteomics study highlighted 121 dysregulated proteins in CBD-treated MCF7 cells compared to the negative controls. We reported the inhibition of topoisomerase II β and α, cullin 1, V-type proton ATPase, and CDK-6 in CBD-treated MCF7 cells for the first time as additional cytotoxic mechanisms of CBD, alongside sabotaged energy production and reduced mitochondrial translation. We observed 91 significantly dysregulated proteins in MCF7 cells treated with the synergistic combination of CBD with SN−38 (CSN−38), compared to the monotherapies. Regulation of telomerase, cell cycle, topoisomerase I, EGFR1, protein metabolism, TP53 regulation of DNA repair, death receptor signalling, and RHO GTPase signalling pathways contributed to the proteome-wide synergistic molecular mechanisms of CSN−38. In conclusion, we identified significant synergistic interactions between CBD and the five important chemotherapeutic drugs and the key molecular pathways of CBD and its synergistic combination with SN−38 in MCF7 cells. Further in vivo and clinical studies are warranted to evaluate the implementation of CBD-based synergistic adjuvant therapies for breast cancer.

## 1. Introduction

Medicinal cannabis and its secondary metabolites have garnered significant attention in recent years, and have been subject to extensive research and scrutiny [1,2,3,4,5,6,7]. From a medicinal and pharmaceutical standpoint, nonpsychoactive phytocannabinoids—such as cannabidiol (CBD)—are the most potentially beneficial components of cannabis’ chemical space [2,8]. CBD has displayed a wide range of pharmacological activities in several preclinical and clinical studies that enabled its potential application in inflammation, cancer, cardiovascular diseases, epilepsy, and neurodegenerative and psychiatric disorders [6,9,10,11,12,13,14,15,16,17,18]. The US Food and Drug Administration (FDA) has approved Epidiolex^®®^—a pure CBD formulation—for use in patients with Lennox–Gastaut syndrome or Dravet syndrome seizures [19]. Sativex^®®^ (2.7 mg THC and 2.5 mg CBD per dose) has also been approved in around 30 countries for pain management in multiple sclerosis [18]. On a global scale, over 40 countries have approved medicinal marijuana/cannabis programmes. However, the Drug Enforcement Administration (DEA) has declared that CBD is a Schedule V banned substance, and its products must contain less than 0.1% ∆^9^-THC to be approved by the FDA [20]. The current legal status of CBD is neither clear nor harmonised worldwide, although there are widespread medicinal, food, or cosmetic products that contain CBD [21,22]. Intriguingly, liquid chromatography coupled with mass spectrometry enables the subnanomolar detection and quantitation of cannabinoids in different biological matrices [23].

CBD displays anticancer potential, blocking cancer initiation, progression, and invasion for various cancer types, including breast, lung, colon, prostate, brain, multiple myeloma, and skin cancers [6,7,9]. CDB, and the endocannabinoid system in general, have long been studied for their potential to treat cancer. Unlike the psychoactive cannabinoids, CBD has a comparatively lower affinity to both CB1 and CB2 receptors [24,25]. Nevertheless, it has been reported to act as a CB1 antagonist in murine brain tissue and vas deferens, or as an inverse agonist in human CB2 receptors [24,26]. Moreover, CBD interaction targets include 5-HT1A, GPR55, and PPAR-γ, in addition to TRPVs [27,28]. The basal respiration rate and ATP production in the gastric cancer cells were decreased by CBD, with suppressed proliferation and tumour growth, in a murine xenograft model [29]. CBD-induced mitochondrial stress in MCF7 cells, with a modulated mitochondrial redox and dynamics, was recently reported [30]. These are all suggestive of CBD’s impacts on the mitochondrial processes and energy production in cancer cells, where holistic multi-omics investigations are warranted in order to understand the underlying mechanisms. Other potential targets for CBD in cancers—such as cyclin-dependent kinases (CDK) and topoisomerases—are not well explored, despite the displayed selective inhibition of topoisomerase II by CBD derivatives such as HU-331 [31]. CBD’s anti-cancer mechanisms, its antiproliferative and pro-apoptotic effects, and its inhibitory effects on cancer metastasis, invasion, and migration were summarised in recent reviews [6,7,9]. CBD is also involved in the regulation of reactive oxygen species (ROS), endoplasmic reticulum (ER) stress, inflammation, and immunological modulation [6].

Breast cancer is the most common cancer type in women worldwide, with the highest regional incidence reported in Australia and New Zealand [32,33]. CBD was found to inhibit oestrogen-receptor-positive (ER+), -negative (ER−) or -triple-negative breast cancer cells (TNBC) in a dose-dependent manner, with relatively low IC_50_ values [6,34,35,36,37,38,39]. This suggests that breast cancer cells are sensitive to CBD, while non-transformed epithelial breast cells (MCF10A) are not significantly affected [10,28,39,40]. Interestingly, the administration of CBD in several clinical trials, as a single dose of 150–900 mg [41,42,43,44,45,46,47,48,49] or 50–1000 mg/day for up to 13 weeks [50,51,52,53], showed overall good tolerability and safety profile, with mild side effects [54]. A variety of mechanisms for CBD’s antiproliferative effects have been reported, including autophagy, cell cycle arrest at the G_1_/S checkpoint, and apoptosis in MCF7, MDA-MB-231, and 4T1 breast cancer cells [34,35,39,55]. Recent studies have found that the antiproliferative effect of CBD in breast cancer cells was independent of endocannabinoid receptors (to some extent), mediated primarily through ROS-induced cell death [34,35,39,56,57]. CBD’s pro-apoptotic actions were attributed to the downregulation of mTOR, AKT, 4EBP1, and cyclin D, as well as the overexpression of PPAR-γ [35,39], where CBD coordinated apoptosis and autophagy via Beclin-1 translocation and cleavage [39]. Furthermore, CBD inhibited the invasive and metastatic nature of aggressive TNBC breast cancer by suppressing the activation of the EGF/EGFR pathway and its downstream targets (AKT and NF-κB), along with Id-1 protein downregulation by ERK and ROS [34,36,37,55,56]. In comparison to daily administration, CBD administered three times per week boosted the longevity of mice while reducing the number of metastases [55,56]. CDB decreased TAM recruitment by downregulating CCL3, GM-CSF, and MIP-2, resulting in an overall reduction in angiogenesis [36]. Nevertheless, more research into the anticancer mechanisms of CBD at the proteome-wide level is needed in order to comprehensively understand and decode its complicated mechanisms to encourage future clinical trials.

Based on the anticancer actions of CBD, it has been suggested that combinations of CBD and chemotherapeutic drugs may overcome cancer resilience by targeting various cancers’ pathophysiological components [58,59]. Synergistic combinations offer the use of lower doses with reduced side effects and curtailed drug resistance [60,61]. For instance, CBD inhibited exosome and microvesicle release, making breast (MDA-MB-231), prostate (PC3), and hepatocellular (HEPG2) cancer cells more sensitive to cisplatin [57]. It also synergistically improved the effects of paclitaxel (PTX) and doxorubicin (DOX) in both MCF7 and MDA-MB-231 breast cancer cells [38]. Moreover, it reduced PTX-induced neurotoxicity via 5-HT1A receptors without affecting nervous system function or chemotherapeutic efficacy [62]. CBD amended DOX-induced cardiomyopathy by regulating the cardiac mitochondrial functions in murine models [63]. Nevertheless, the synergistic interactions of CBD with chemotherapeutic drugs have not been thoroughly investigated. In particular, limited data are available on effective doses and drug interactions, and further research is required [64,65]. In addition, the utilisation of different synergy metrics is fundamental, as there is no consensus on a gold standard synergy quantification model [66,67,68,69].

In the present study, MCF7 cell line was selected, as it is the most studied human breast cancer cell line [70]. In addition, breast cancer chemotherapeutic agents with different modes of action—taxane microtubule stabilisers (docetaxel, paclitaxel), anthracyclines (doxorubicin), microtubule-disruptive vinca-related alkaloids (vinorelbine), and topoisomerase I inhibitors (irinotecan metabolite = SN−38)—and their CBD combinations were studied. These chemotherapeutic agents represent the most common treatment options for stage I–IV breast cancer [71], along with other therapies, including hormonal therapy, radiotherapy, or surgery. We aimed to evaluate the synergistic interactions of CBD with chemotherapeutic drugs using different synergy quantitation metrics. The apoptotic profiles for the synergistic combinations were evaluated using flow cytometry. Furthermore, a shotgun proteomics study was conducted to decipher the proteome-level cytotoxic and synergistic mechanisms of CBD, and the most prominent (SN−38) synergistic combination.

## 2. Results and Discussion

### 2.1. Synergy Quantification of CBD and Standard Chemotherapeutic Drugs in MCF7 Human Breast Adenocarcinoma Cells

**Figure 1:**
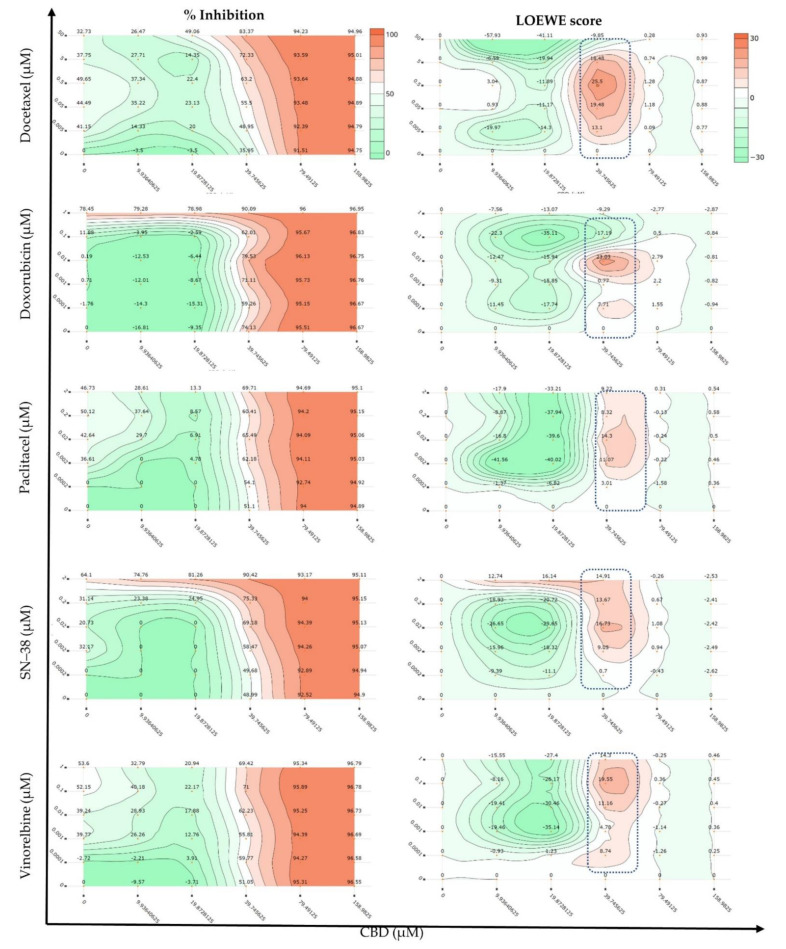
Dose–response and Loewe synergy quantitation of cannabidiol (CBD) combinations with the chemotherapeutic drugs against MCF7 cells in a checkerboard assay (*n* = 3).

Since there is no agreement on a benchmark synergy model [69] to decipher the complex interactions between drugs, the exploration of different synergy models is advised. Therefore, multiple synergy quantification metrics were implemented to gain a comprehensive understanding of the potential synergistic interactions between CBD and the selected chemotherapeutic drugs. The combination index (CI) model can be utilised for pairwise or higher order drug combinations in constant and non-constant ratio combinations [72]. This model was used to quantify the cytotoxic interactions against MCF7 cells after 72 h of treatment. The CI < 1 and CI > 1 indicate synergism and antagonism, respectively, whereas additivity is indicated by CI = 1 [72]. CBD and the chemotherapeutic drugs were combined in molar ratios from 1431–18:1, respectively (Table 1). CompuSyn-calculated CI values at 50, 75, 90, 95, and 97% inhibitory concentrations are reported in Table 1. Each combination was denoted by an ID (e.g., CDOX19), where the last two digits indicate the corresponding molar ratio of CBD and DOX, as reported in Table 1. In addition, a checkerboard assay was used to combine the chemotherapeutic drugs with CBD in 1:10 and 1:2 serial dilutions, respectively (Figure 1). Therefore, synergy quantification in the Loewe, ZIP, BLISS, HSA, and S synergy score models along the combination sensitivity score (CSS) were scrutinised over a broader range of anticancer concentrations in order to comprehensively understand the synergistic interactions of CBD with the chemotherapeutic drugs against MCF7 cells.

**Figure 2:**
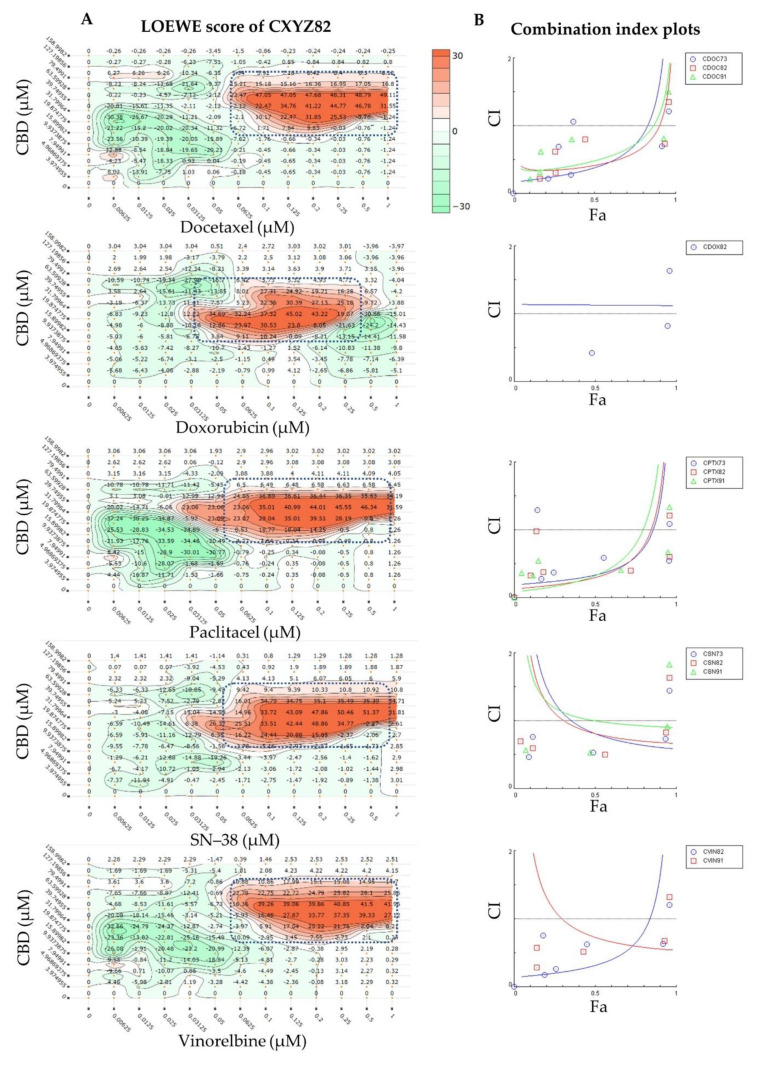
Most synergistic chemotherapeutic cannabidiol (CBD) combinations against MCF7 cells: (**A**) Loewe synergy quantitation of selected synergistic CBD combinations with standard chemotherapeutic agents (molar ratio of 636:1). (**B**) Combination index plots of the most synergistic combinations. Fa = fraction affected (% cell growth inhibition, where 1 = 100%).

A strong synergy was observed in different synergy models for the CDB combined with SN−38 (CSN91, CSN82, and CSN73) and vinorelbine (CVIN91). These combinations, unlike CPTX or CDOC, showed increased synergy in the CI model, with increased cell growth inhibition (Figure 2B), which is favoured for any anticancer treatment, where any antagonistic behaviour at a low fraction affected (Fa < 0.5 = 50% growth inhibition) will not be alarming. CBD and SN−38 combinations with a molar ratio of 1431, 636, or 371 to 1 displayed CI values of 0.9–0.93, 0.67–0.81, and 0.60–0.81, respectively, at the IC_50_ to IC_97_ modelled inhibitory concentrations. These combinations displayed synergistic scores in all DrugComb synergy models tested (Loewe, Bliss, HSA, ZIP, and S) when the same response data were imported to the DrugComb webserver. Moreover, CVIN91 (molar ratio of 1431:1 of CBD:VIN) showed a CI value of 0.55–0.73, with positive synergy scores in all DrugComb models. In addition, CVIN82 displayed synergy in all DrugComb models, with synergistic CI values of 0.32 and 0.65 at IC_50_ and IC_75_, respectively. Interestingly, all CBD chemotherapeutic combinations at a molar ratio of 636:1 were synergistic in the Loewe, Bliss, HSA, ZIP, and S models, in agreement with the CI model at different inhibitory concentrations. Nevertheless, discording interactions were observed for the Bliss model of DOC and the CI model of DOX (Figure 2B), together with the CI values at some inhibitory concentrations for DOC, DOX, PTX, and VIN (Table 1). Both checkerboard and CI design data reanalysed in DrugComb concurred on the captured synergy of around 39 µM CBD with various concentrations of different drugs (Figure 1 and Figure 2). All of the IC_50_ values for the different combinations and monotherapies in the CI design are listed in Table S1. Discording CompuSyn- and GraphPad-calculated IC_50_ values were observed—particularly for the standard chemotherapies, despite the same data having been implemented. These findings should be considered while using only CompuSyn for synergy evaluation.

**Figure 3:**
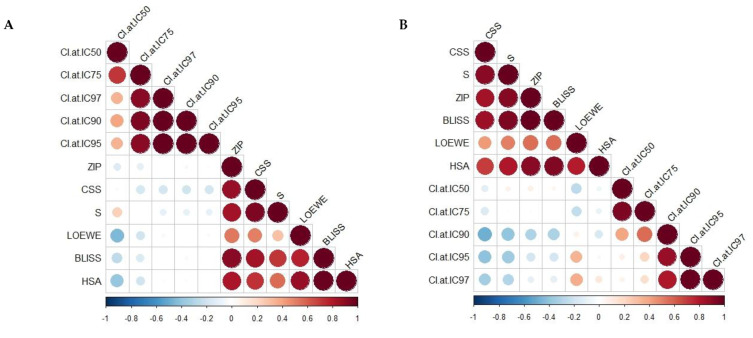
Correlation matrix of different synergy metrics and combination sensitivity scores (CSSs) using (**A**) Pearson and (**B**) Spearman correlation coefficients. CI = combination index derived from Chou–Talalay model; IC = inhibitory concentration killing the corresponding percentage of MCF7 cells. Colour indicates the correlation coefficient along with negative correlations in blue and positive correlations in red, with the size being directly proportional to statistical confidence based on *p-*values.

Figure 3 and Table S2 show the Pearson and Spearman correlation matrices as linear and nonparametric rank correlation measurements, respectively, for different synergy quantification metrics and CSS. A non-significant weak correlation of the Loewe, Bliss, HSA, S, and Zip models with the CI model was indicated by the Pearson and Spearman correlation coefficients, except for the Loewe and HSA models, which were weakly and negatively correlated with the CI model at IC_50_ (Pearson r −0.45, −0.39, respectively; *p* < 0.05). The same trend was observed for the CSS and S models’ Spearman correlation with the CI model at high inhibitory concentrations (IC_90_–IC_97_). The negative correlation displayed the different scaling, where the synergistic score of the CI model should be < 0, and that for the DrugComb synergy metrics should be > 0. However, moderate-to-strong Pearson and Spearman correlations were observed among the DrugComb synergy models and CSS (0.7–0.99, *p* < 0.05), except for Loewe with the CSS, S, and ZIP models (Pearson r = 0.5, 0.29, and 0.52, respectively; *p* < 0.05) and HSA with the S synergy model (Pearson r = 0.56). Taken together, different synergistic interactions could have been drawn, implementing different models—particularly the CI model versus others. Notably, various synergy metrics—including Loewe, ZIP, HSA, and Bliss—displayed a moderate correlation with strong disagreement instances when calculated for the O’Neil anticancer combination dataset (22,737 unique combinations) [67,69,73]. Moreover, substantial disagreements were reported when correlating synergy scores originating from different datasets [67]. Thus, the selection of synergistic doses needs to be validated against different models.

Unlike synergy, which captures the drug interactions, the combination sensitivity calculates the efficacy of the combined drugs, and its negligence may lead to biased synergistic combinations [74]. The relative IC_50_ value and the area under the drug combination dose-response curve were utilised to derive the CSS, which was presented as a robust metric for efficacy quantification of drug combinations [75]. Table 1 summarises the combined sensitivity scores for the combinations of CBD with the chemotherapeutic drugs tested via checkerboard assay, or upon reprocessing of CI model data via DrugComb and their aggregated data (CI to DC). Notably, promising CSS values were observed for the synergistic combinations against different study designs. The highest CSS (>70) values were displayed for SN−38 and DOC combinations in the checkerboard assay, and DOX in the CI to DrugComb models. Considerably elevated CSS values were noticed for DOX combinations (CDOX19, CDOX28, CDOX37, CDOX64, and CDOX73), along with positive synergy scores in the ZIP, Bliss, and HSA synergy metrics, despite the marginally synergistic to weak antagonistic CI values (Table 1).

Previously, synergistic CBD interactions with DOX and PTX were studied against MCF7 and MDA-MB-231 cells in the CI model. A limited range of responses (total of nine doses), resulting from mixing three fixed doses of CBD with DOX (20, 1, and 0.1 uM) and PTX (0.5, 0.1, and 0.01 uM), were examined [38]. The molar ratio ranges from 20–2000 and 0.5–200 of CBD to 1 of PTX or DOX, respectively, were also explored in MCF7 cells [38]. Our findings are partially in agreement with the latter study in some synergy models, but we considered a broader range of responses (54 and 36 for our CI and checkerboard designs, respectively) in different synergy models, together with CSS calculations. Interestingly, CBD was previously reported to ameliorate DOX-induced cardiomyopathy via modulating cardiac mitochondrial functions such as complex I and complex II activities in mice [63,76]. In addition, CBD—or its combination with Δ^9^THC—reduced the PTX-induced neuropathic pain in mice without affecting the nervous system function or PTX efficacy [62,77]. Collectively, the cardioprotective and pain relief effects of CBD, along with its synergistic interactions with DOX or PTX, warrant further studies for its potential implementation in clinical practice.

CBD has been reported to potentiate DOC-inhibitory effects in LNCaP and DU-145 xenografts in vivo, and against LNCaP, 22RV1, DU-145, and PC-3 cells in vitro [78]. Interestingly, the coadministration of medicinal cannabis herbal tea did not affect the pharmacokinetics of irinotecan (and its SN−38 metabolite) and DOC [79]. Nevertheless, the potential synergistic interactions of CBD with DOC, VIN, and SN−38 have not previously been studied. The synergistic interactions of DOC, VIN, and SN−38 with CBD found in the current study require further in vivo investigations to evaluate the enhanced chemotherapeutic effects where dose reduction of chemotherapy may be expected.

In the present study, selected doses of combined CBD and chemotherapeutic drugs were validated against different models, including CI and DrugComb synergy metrics, with at least 90% inhibition of MCF7 cells. For DrugComb synergy models, both checkerboard design data and the combined dose responses of different combinations (CXYZ19 to CXYZ91) were considered for the selection of the synergistic doses. These synergistic doses were further utilised in the subsequent flow cytometry and shotgun proteomics studies.

### 2.2. Flow Cytometric Analyses of Apoptosis in MCF7 Human Breast Adenocarcinoma Cells Using Annexin V-CF Blue and 7-Aminoactinomycin D (7AAD)

**Figure 4:**
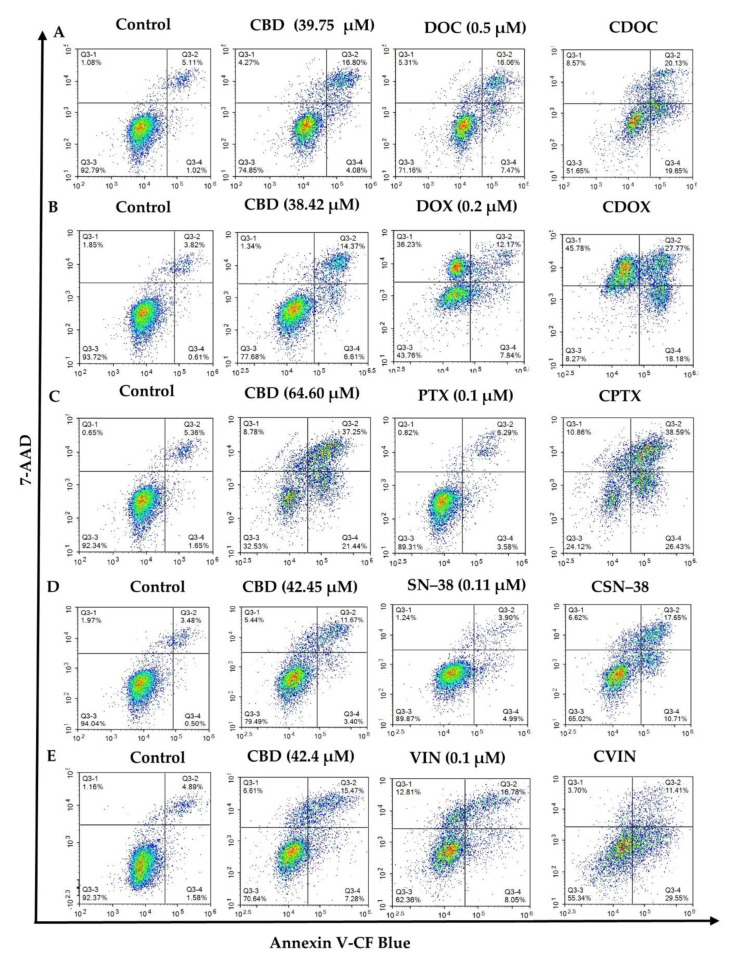
Flow cytometric assessment of the apoptotic profiles of MCF7 cells after 24 h of treatment with cannabidiol, chemotherapeutic drugs, and their synergistic combinations (**A–E**). The images are representative of three separate experiments. CBD = cannabidiol: DOC = docetaxel: DOX = doxorubicin: PTX = paclitaxel: SN−38: VIN = vinorelbine. (**A**) CDOC = CBD + DOC, (**B**) CDOX = CBD + DOX, (**C**) CPTX = CBD + PTX, (**D**) CSN–38 = CBD + SN–38, (**E**) CVIN = CBD + VIN. Cells were treated with the monotherapies, combinations, and vehicle control (0.5% DMSO), and detected using antibodies against Annexin V-CF Blue and the reporter 7AAD after 24 h of treatment. Raw data are available in Table S3.

**Figure 5:**
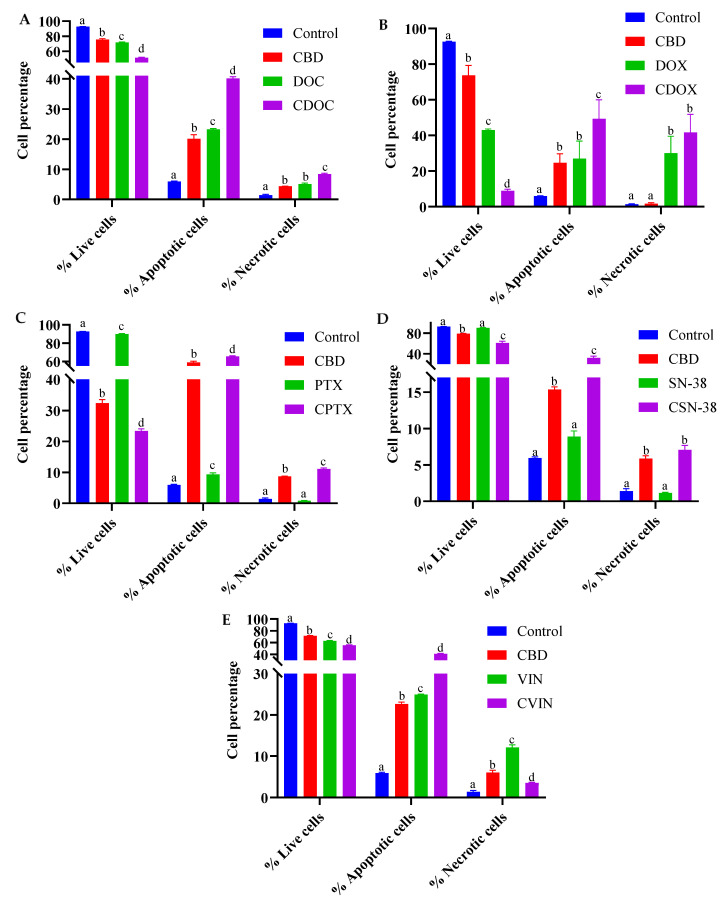
Cell percentage analysis of MCF7 cells after 24 h of treatment with cannabidiol, chemotherapeutic drugs, and their synergistic combinations (**A-E**). CBD = cannabidiol: DOC = docetaxel: DOX = doxorubicin: PTX = paclitaxel: SN−38: VIN = vinorelbine. (**A**) CDOC = CBD (39.75 µM) + DOC (0.5 µM), (**B**) CDOX = CBD (38.42 µM) + DOX (0.2 µM), (**C**) CPTX = CBD (64.60 µM) + PTX (0.1 µM), (**D**) CSN−38 = CBD (42.45 µM) + SN−38 (0.11 µM), (**E**) CVIN = CBD (42.4 µM) + VIN (0.1 µM). Superscript letters indicate statistical significance derived from two-way ANOVA and Tukey’s multiple comparisons within the same cell group, where different letters are statistically significant at *p* < 0.05 (*n* = 3). Raw data are available in Table S3.

The apoptotic profiles of MCF7 cells were evaluated using flow cytometry after 24 h of treatment with CBD, chemotherapeutic agents, or their combinations. The chemotherapeutic drugs (DOC, DOX, PTX, SN38, and VIN) and their most synergistic combinations with CBD were evaluated at the doses indicated in Table 1. A concurrent evaluation of apoptotic and necrotic MCF7 cells was carried out to detect whether the most synergistic combination had any enhanced apoptotic effects compared to monotherapy (Table S3). After 24 h, significant differences in the live, early or late apoptotic, total apoptotic, and necrotic cell populations were observed in the negative controls, monotherapies, and combined treatments in pairwise comparisons via two-way ANOVA and Tukey’s test for multiple comparisons correction (*p* < 0.05, *n* = 3) (Figure 4, Figure 5 and Figure S1, and Table S3).

CBD stimulated programmed cell death in the MDA-MB-231 breast cancer cells, with regulated crosstalk between apoptosis and autophagy [39]. In both the T-47D and MDA-MB-231 breast cancer cells, CBD treatment coordinated an interplay between PPAR-γ, mTOR, and cyclin D1 that favours apoptotic induction [35]. In the present study, CBD alone (38.42–64.6 µM) significantly reduced the live population of MCF7 cells from around 92% to 78.74–32.37% (*p* < 0.001), in a dose-dependent manner. The total apoptotic MCF7 cells were significantly increased from 5.94% to 15.38–58.94% by CBD in different concentrations (*p* < 0.0001) compared to the negative controls (Figure 4B and Figure 5B; *p* = 0.0058 for 38.42 µM CBD). However, different CBD doses significantly increased necrosis in MCF7 cells (*p* < 0.01), from 1.4% to 8.69%, with the highest CBD concentration (64.60 µM). Altogether, a significant 3–11-fold increase in the number of apoptotic cells was observed for different CBD doses in MCF7 cells compared to the untreated control cells, with a decreased living cell percentage and a slight increase in the number of necrotic cells (0.29–7.29%).

Similarly, a significant reduction in the number of live cells, and increased numbers of apoptotic and necrotic cells, were observed for DOC (0.5 µM) and VIN (0.1 µM) as monotherapies (*p* < 0.0001 in comparison to the untreated MCF7 cells; Figure 4A,E and Figure 5A,E). PTX (0.1 µM) showed the same trend, except with a non-significant difference for necrotic cells (*p* = 0.58). A substantial increase in necrotic cells (~20-fold change compared to the controls; *p* < 0.0001) was observed for 0.2 µM DOX (Figure 4B and Figure 5B). SN−38 showed a weak decrease in the number of living cells, but its effect on apoptosis was not statistically significant at the currently selected dose of 0.11 µM (Figure 4D and Figure 5D).

A significant decrease in the live MCF7 cell population, with enhanced apoptosis, was observed for all of the synergistic CDB combinations, compared to monotherapies or negative controls (*p* < 0.0001), except for apoptotic cell percentage of CDOX compared to CBD or DOX, which was slightly higher, with *p* = 0.0003 and 0.001, respectively (Figure 4, Figure 5 and Figure S1, Table S3). To this end, CBD enhanced the apoptotic activity of the studied chemotherapeutic drugs, which consequently contributed to the overall synergistic activity. For the following section, a shotgun proteomics discovery study was conducted to decipher the cytotoxic molecular mechanisms of CBD, and its possible synergistic mechanisms with SN−38 against MCF7 cells.

### 2.3. Bottom-Up Label-Free Quantification Proteomics Study of MCF7 Cell Lysates after Treatment with CBD or Its SN−38 Synergistic Combination

**Figure 6:**
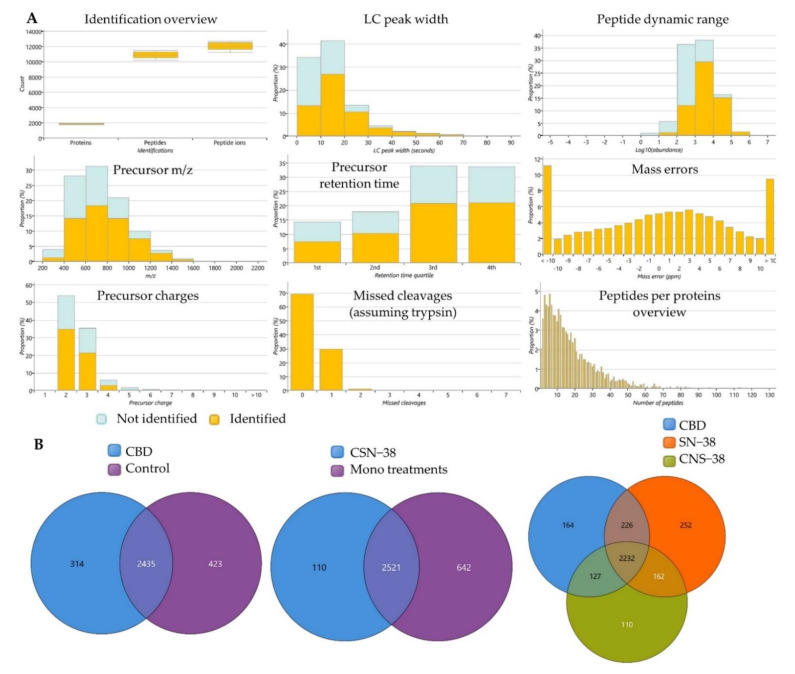
Label-free quantification proteomics study of cannabidiol (CBD) synergistic combination with SN−38 in MCF7 cells. (**A**) Overview of quality control metrics for the label-free quantification proteomics study showing peptides and protein counts, peptide charges, missed cleavage (approximately 70% of the identified peptides showed zero missed cleavage), most of the peptides eluted in the 3rd and 4th quartiles of the LC run, peak width, identified peptides per proteins, and mass errors proportions in the identified peptides. (**B**) Venn diagrams of the overlapped identified proteins’ counts in the differently treated groups. CSN−38 = synergistic combination of CBD and SN−38; control = vehicle (0.5% DMSO)-treated MCF7 cells.

A shotgun proteomics study of MCF7 cells treated with 42.45 µM CBD, 0.11 µM SN−38, and their synergistic combination (CSN−38) was performed, with relative quantification, implementing the Hi-N method. The expressed proteins in CBD-treated MCF7 cells were analysed in pairwise comparisons to the control group to trace for the difference in the proteome-wide dysregulated expressions that may be associated with the cytotoxic effects of CBD. To highlight the possible CSN−38 synergistic mechanisms, the differentially expressed proteins in the combination-treated cells were analysed in pairwise comparison to the monotherapies. Among the total 3569 identified proteins, 1256 and 758 were found to be significantly different in CBD vs. Control and CSN−38 vs. monotherapies, respectively, with *p* and Q values ≤ 0.05 (Q-values are the adjusted *p*-values, and are derived based on the optimised false discovery rate (FDR) approach). Figure 6 displays the quality control metrics and overlapped proteins among different experimental groups. Decent tryptic digestion could be speculated with minimal missed cleavages, and most of the peptides were eluted in the 3rd and 4th quartiles of the LC run. In addition, the peptides with an absolute mass error of 20 ppm were disregarded based on the mass error distribution of the identified peptides (Figure 6A). The peptide counts, unique peptide counts, m/z, confidence scores, statistics, and fold change (FC) calculations are listed in Supplementary File 2.

#### 2.3.1. Proteome-Wide Elucidation of the Cytotoxic Effects of CBD in MCF7 Cells: Pilot Shotgun Proteomics Study

The differentially expressed proteins in CBD-treated MCF7 cells, compared to the controls, were selected implementing *p*- and Q-values of ≤ 0.01 alongside an absolute FC ≥ 2 (absolute log2 FC ≥ 1) cutoff. A total of 25 upregulated and 96 downregulated proteins were identified in CBD-treated cells (Table 2 and Table 3, and Supplementary File 2 (CBD vs. control sheet)). These 121 dysregulated proteins represent the proteome-level variance acquired upon CBD treatment. Therefore, these proteins may reflect the possible underlying mechanisms of its cytotoxic effects in MCF7 cells.

**Figure 7:**
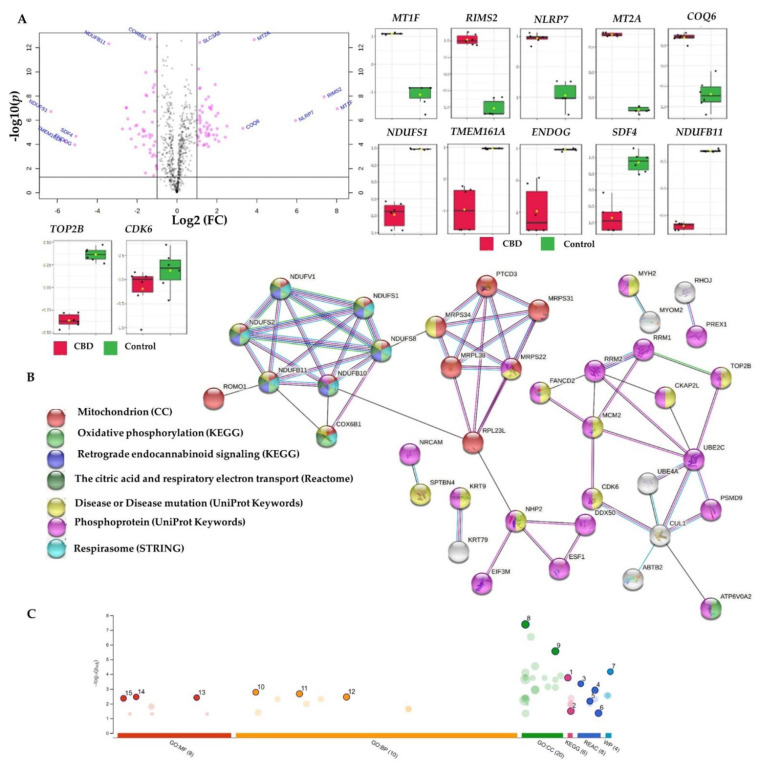
Differentially expressed proteins in cannabidiol (CBD)-treated MCF7 cells compared to controls, and the corresponding overrepresented pathways. (**A**) Volcano plot with an absolute log2 fold change ≥ 1 and *p*-value ≤ 0.05 cutoff, for the identified proteins in CBD-treated cells, together with selected proteins’ expression summary compared to the controls. (**B**) STRING network of the 121 differentially expressed proteins (fold change ≥ 2; *p* and Q values ≤ 0.01) in the cannabidiol-treated MCF7 cells compared to the controls. The minimum required interaction score was 0.40 (medium confidence), and red, green, blue, purple, light blue, and black interaction lines indicate the presence of fusion, neighbourhood, co-occurrence, experimental, database, and co-expression evidence, respectively. The disconnected nodes were hidden in the network. (**C**) Overrepresented pathways using g:Profiler for the differentially expressed proteins (fold change ≥ 2; *p* and Q values ≤ 0.01) in the cannabidiol-treated MCF7 cells compared to the controls (1 = oxidative phosphorylation; 2 = retrograde endocannabinoid signalling; 3 = complex I biogenesis; 4 = respiratory electron transport; 5 = mitochondrial translation initiation; 6 = the citric acid (TCA) cycle and respiratory electron transport; 7 = mitochondrial complex I assembly model OXPHOS system; 8 = mitochondrial inner membrane; 9 = mitochondrial protein-containing complex; 10 = mitochondrial electron transport; NADH to ubiquinone; 11 = aerobic electron transport chain; 12 = mitochondrial ATP synthesis-coupled electron transport; 13 = NADH dehydrogenase (quinone) activity; 14 = NADH dehydrogenase (ubiquinone) activity; 15 = NADH dehydrogenase activity). CC: cellular components; MF: molecular function; BP: biological processes; REAC: Reactome database; WP: Wiki Pathways.

STRING [80], Reactome [81], g:Profiler [82], and IMPaLA [83] overrepresentation analyses identified a subset of downregulated proteins encoding genes such as *NDUFV1*, *NDUFB11*, *NDUFB10*, *NDUFS1*, *NDUFS2*, and *NDUFS8*. These proteins were involved in complex I biogenesis, respiratory electron transport, mitochondrial ATP synthesis, the citric acid (TCA) cycle, oxidative phosphorylation, and retrograde endocannabinoid signalling, together with *COX6B* and *ATP6V0A2*, for all of the aforementioned processes except for retrograde endocannabinoid signalling (Table 3, Figure 7, and Supplementary File 2, ORA_Enrich. of CBD vs. C sheet).

Aerobic respiration occurs exclusively in the mitochondria of the eukaryotic cells and starts with glycolysis and the TCA cycle; followed by complexes I–IV, which form the electron transport chains (ETC) within the inner membrane of the mitochondria [84,85]. Complex I is the largest complex of the mitochondrial ETC, offering approximately 40% contribution of the redox-driven proton pump necessary for ATP production in the mitochondria [86]. Notably, complex I is required for biosynthesis and redox regulation during cancer cell growth, resistance to cell death, and metastasis [86]. CBD’s paradoxical regulatory effects on mitochondrial functions were recently reviewed in brain tissue, isolated mitochondria, and hippocampal cells [87]. CBD (4 µM) was previously found to significantly undermine the basal respiration rate and ATP production in gastric cancer cells with suppressed proliferation and tumour growth in xenografted mice [29]. A similar trend was observed in the HCT116 and DLD-1 colorectal cancer cells (CRC) with Noxa- and ROS-dependent apoptosis [88].

V-ATPase inhibitors were found to facilitate the control of the tumour microenvironment, with reduced tumour acidity and metastasis, along with the prevention of chemoresistance [89].

In light of the aforementioned downregulated proteins and their involvement in the biological processes, CBD-mediated apoptosis and cytotoxicity in MCF7 cells may involve a decreased mitochondrial energy production via the downregulation of complex I- and IV-related proteins (FC = −8.44 to −1.15). In addition, CBD can potentially reduce the tumour acidic microenvironment and metastasis by downregulating V-type proton ATPase (FC = −1.16). Here, CBD was reported for the first time among V-ATPase inhibitors.

In our study, CBD repressed the mitochondrial translation in MCF7 cells. Briefly, the initiation, elongation, and termination of the mitochondrial translation were also over-represented in recruiting the 121 dysregulated proteins in different platforms (Table 3, Figure 7, and Supplementary File 2). The downregulated *PTCD3-*, *MRPS22-*, *MRPS34-*, *MRPL38-*, *MRPS31-*, and *MRPL23*-encoded proteins (FC = −2.27 to −1.57) were involved in the inhibited mitochondrial translation. Mitochondrial translation inhibition has been suggested as a therapeutic strategy for ovarian cancer [90] and human acute myeloid leukemia [91]. Altogether, CBD mediated the mitochondrial power outage alongside the inhibited mitochondrial translation in MCF7 breast cancer cells.

Expression dysregulation was observed for proteins involved in DNA synthesis, the cell cycle, mitosis checkpoints, and G_1_/S transition, such as the upregulated *CDC25B-*, *NHP2-*, and *PSMD9*-encoded proteins and the downregulated *RRM2-*, *CUL1-*, *MCM2-*, *UBE2C-*, and *CDK6-*encoded proteins (Table 2 and Table 3, Figure 7, and Supplementary File 2). In addition, D-type cyclins and their binding partner kinases are important regulators of cell cycle progression, tumour development, and proliferation in breast cancer and normal breast epithelial cells [92]. Multiple mitogenic signalling pathways regulate and influence CDK4/6 and cyclin D activity, including oestrogen receptors and receptor tyrosine kinase (RTK), as well as the PI3K–AKT–mTOR or RAS–RAF–MEK–ERK pathways [92]. Of note, CDK6 knockdown was shown to overcome the abemaciclib resistance in MCF7 cells [93]. In this context, CBD-mediated inhibition of CDK-6 in MCF7 cells (FC = −1.96) could be utilised to enhance the sensitivity of CDK4/6 inhibitors such as abemaciclib, palbociclib, and ribociclib for the treatment of hormone-receptor-positive breast cancer. Additionally, the downregulated cullin 1 (FC = −1.97), *CUL1*-encoded protein may be correlated with curtailed proliferation, migration, and invasion of breast cancer [94,95,96]. Furthermore, CBD-downregulated minichromosome maintenance proteins (MCM), such as the DNA replication licensing factor MCM2, may elucidate the effects of CBD on replication and cell cycle development, while a recent meta-analysis postulated the overexpression of MCM2-7 as a predictive biomarker of poor cancer prognosis [97]. Similarly, a growing body of evidence supports the oncogenic potential of the ubiquitin-conjugating enzyme E2C (*UBE2C*)’s overexpression in breast cancer, which is linked to the loss of *BRCA1* function and induced chemical resistance in MCF7 and MDA-MB-231 cells [98]. Notably, the enhanced effects of radiation, doxorubicin, tamoxifen, and letrozole were observed in breast cancer cells upon the downregulation of *UBE2C* [99]. Taken together, the CBD-mediated downregulation of *MCM2-* and *UBE2C*-encoded proteins contributed to the overall cytotoxicity of CBD, along with the suppressed proliferation, migration, and invasion via the declined expression of cyclin-dependent kinase 6 (*CDK6*) and cullin 1 in MCF7 cells.

Topoisomerase II (TOP2) inhibitors are potent chemotherapeutic drugs that regulate DNA replication [100]. DNA Topoisomerase II*β* and *α* (*TOP2B* and *TOP2A*) were significantly downregulated in CBD-treated MCF7 cells compared to the controls, with FC = −1.25 and −0.72, respectively (Table 3 and Supplementary File 2). Previously, CBD derivatives such as HU-331 displayed selective inhibition to topoisomerase II isoforms via non-competitive inhibition of their ATPase activity [31]. Here, we report the inhibition of TOP2 isoforms in CBD-treated MCF7 cells for the first time, as an additional cytotoxic mechanism, along with the aforementioned downregulation of *CDK-6-*, *MCM2-*, and *UBE2C*-encoded proteins. In addition, the mitochondria-related mechanisms were underlined, including sabotaged energy production and mitochondrial translation.

#### 2.3.2. Proteome-Wide Elucidation of Synergistic Mechanisms of SN−38 Synergistic Combination with CBD in MCF7 Cells: Pilot Shotgun Proteomics Study

The differentially expressed proteins in the synergistic CBD and SN−38 combination-treated cells, compared to the monotherapies, were selected implementing *p*- and Q-values of ≤ 0.01 alongside a maximum FC ≥ 2 (absolute log2 FC ≥ 1) cutoff. A total of 5 upregulated and 31 downregulated proteins were identified in CBD-treated cells. These 36 dysregulated proteins represent the proteome-level variance acquired upon CSN−38 treatment (Table 4). Therefore, they may reflect the possible underlying synergistic mechanisms of action against MCF7 breast adenocarcinoma cells. Relaxed *p*- and Q-values of ≤ 0.05, together with the same FC cutoff, identified 91 dysregulated proteins recruited in further pathway analyses, where no significant pathway enrichment was identified implementing the 36 most significant proteins.

**Figure 8:**
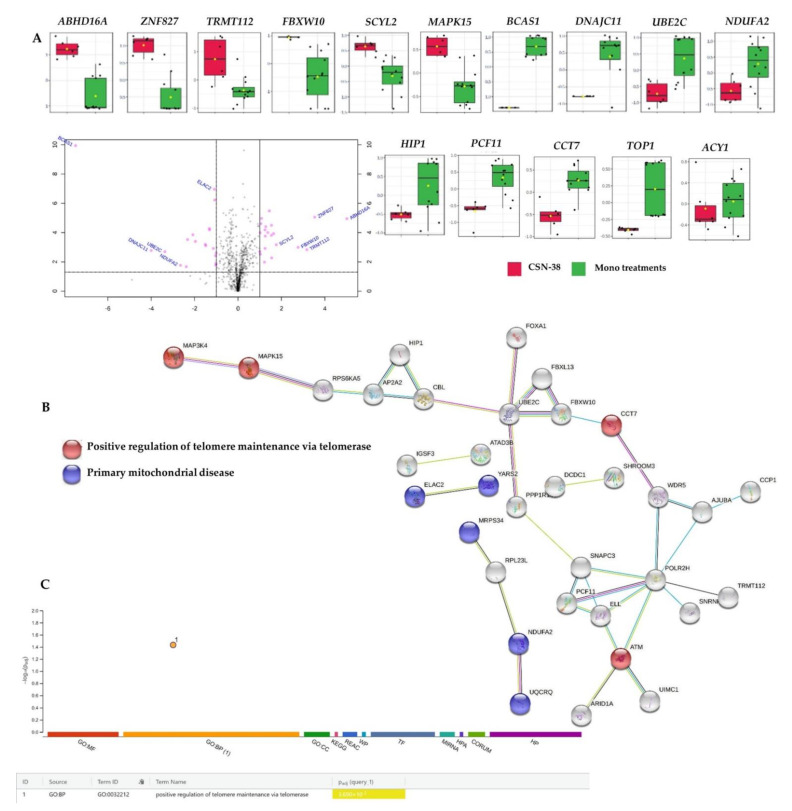
Differentially expressed proteins in synergistic CBD and SN−38 combination-treated MCF7 cells (CSN−38) compared to the monotherapies, and the corresponding overrepresented pathways. (**A**) Volcano plot with an absolute log2 fold change ≥ 1 and *p*-value ≤ 0.05 cutoff of the identified proteins in the CSN−38-treated cells, together with the selected proteins’ expression summary compared to the monotherapies. (**B**) STRING network of the 91 differentially expressed proteins (fold change ≥ 2; *p* and Q values ≤ 0.05). The minimum required interaction score was 0.40 (medium confidence), and red, green, blue, purple, light blue, and black interaction lines indicate the presence of fusion, neighbourhood, co-occurrence, experimental, database, and co-expression evidence, respectively. The disconnected nodes were hidden in the network. (**C**) Overrepresented pathways using g:Profiler for the differentially expressed proteins (fold change ≥ 2; *p* and Q values ≤ 0.05).

A subset of downregulated proteins encoding genes such as *CCT7*, *ATM*, and *MAP3K4*, alongside the overexpressed *MAPK15* involved in the positive regulation of telomere maintenance by telomerase, was identified upon the recruitment of the 91 dysregulated proteins (Supplementary File 2, CSN38 vs. Monotherapies sheet) in STRING [80] and g:Profiler [82] overrepresentation analyses (Figure 8).

We observed overexpressed chaperonin-containing TCP-1s (CCTs), such as *CCT7*, which was shown to be involved in breast cancer progression, associated with poor overall survival in breast cancer patients, and was recently speculated as a potential breast cancer therapeutic target and valuable prognostic marker [101]. In addition, ATM serine/threonine kinase encoded by *ATM* is involved in DNA repair, cell cycle arrest, and apoptosis as activated and recruited upon DNA double-strand breaks (DSBs) [102,103]. *ATM* gene mutations result in ataxia–telangiectasia, where disrupted recognition and repairs of DNA double-strand breaks (DSBs) take place, with increased risk of cancer [104,105]. CSN−38 significantly downregulated both T-complex protein 1 subunit eta (*CCT7*) and *ATM* in MCF7 cells (−1.78 and −2.84 FC, respectively) compared to the monotherapies, where impaired DNA DSB repairs and tumour progression could be anticipated through the synergism of CBD and SN−38.

Mitogen-activated protein kinase 4 (*MAP3K4*) was also downregulated and was highlighted as a potential radiation response biomarker in both post- and preoperative settings in breast cancer patients [106]. Unlike healthy tissue, tumours increase extracellular acidification as cancer cells ferment glucose with increased lactate production, and maintain the same level of mitochondrial respiration even in the presence of oxygen, which was hypothesized by Otto Warburg as a hallmark of cancer [107,108]. Interestingly, the *MAP3K4* downregulation inhibited the Warburg effect by regulating lactate secretion and lactate receptor expression through the HER2/HER3 signalling pathways in MCF7 cells [109]. In our study, CSN−38 significantly decreased the expression of *MAP3K4* (FC −1.15, Q = 0.025; Supplementary File 2, CSN38 vs. Monotherapies sheet), which might inhibit the migration and extracellular acidity of MCF7 cells. In addition to *MAP3K4*, *MAPK15* was also involved in the MAPK signalling pathway and was overexpressed in the CSN38-treated cells compared to the monotherapies. Increased migration of breast and lung cancer cells was reported earlier upon the downregulation of *MAPK15* (alias ERK8), which acts as a negative regulator of cell migration and O-glycosylation of N-acetylgalactosamine (GalNAc) [110]. O-GalNAc glycosylation in the endoplasmic reticulum was reported as a driving mechanism of cancer invasiveness [111]. Based on the current findings, the CSN−38 treatment might inhibit the invasiveness and migration of MCF7 cells by increasing *MAPK15* expression (FC 1.1).

Several proteins involved in the cell cycle, EGFR1, metabolism of proteins, TP53 regulation of DNA repair, death receptor signalling, and RHO GTPase signalling pathways were significantly over-represented or enriched (*p* ≤ 0.05) in IMPaLA or Reactome analyses upon recruiting the 91 dysregulated proteins in the combination-treated MCF7 cells. However, a high false discovery rate or Q value ≥ 0.05 was observed for these pathways (Supplementary File 2, ORA_Enrich of CSN38 vs. Monotherapies sheet). All of the involved proteins identified in Reactome-enriched pathways were downregulated (encoded by *ELL*, *ATM*, *POLR2H*, *PCF11*, *SNAPC3*, *ABR*, *PREX1*, *IRAK1*, *SPPL2A*, *NGEF*, *ITGAL*, *KLKB1*, *TMEM87A*, *ELAC2*, *UBE2C*, *ARID1A*, *NUP93*, *WDR5*, *ZNF546*, *ZNF521*, *CCT7*, *ACY1*, *AJUBA*, *NRCAM*, and *CBL*), except for *FBXW10-*encoded protein, which was overexpressed.

The most downregulated proteins in the combination-treated MCF7 cells were breast carcinoma-amplified sequence 1, DnaJ homologue subfamily C member 11, NADH:ubiquinone oxidoreductase subunit A2, and ubiquitin-conjugating enzyme E2 C (FC −27.75, −24.86, −5.01, and −4.84, respectively), compared to the monotherapies. The synergistic combination significantly diminished the expression of breast carcinoma-amplified sequence 1—a breast cancer oncogenic protein that is linked to mammary tumourigenesis [112]. Complex I-related proteins (*NDUFA2*) and *UBE2C*-encoded protein are discussed in Section 2.3.1. DnaJ homologue subfamily C member 11 is a heat shock protein, recently found to be upregulated in breast cancers such as basal, luminal B, and HER2 breast cancer subtypes, and downregulated in other tumour types; however, its functional role in breast cancer has not yet been deciphered [113,114].

Three downregulated proteins encoded by *ABR*, *PREX1*, and *NGEF* (FC −1.84, −1.06 and −1.0, respectively) were involved in death receptor signalling, p75NTR receptor-mediated signalling (NGFR), and cell death signalling via NRAGE, NRIF, and NADE, as identified in Reactome enrichment analysis (*p*-values 2.82 × 10^−3^, 8.25 × 10^−4^ and 4.51 × 10^−3^, respectively). *IRAK1*-encoded interleukin 1 receptor-associated kinase 1 was also significantly involved in death receptor signalling, p75NTR receptor-mediated signalling, and p75NTR signals through NF-κB, as identified by Reactome and IMPaLA. *IRAK1* silencing in MCF7 cells was previously reported to decrease invasion, proliferation, and migration, with enhanced sensitivity to PTX [115]. Aggressive growth, PTX resistance, and metastasis were also reported in triple-negative breast cancer cells mediated via *IRAK1* overexpression [116]. Recently, a large study including 1085 breast cancer patients showed a positive correlation of decreased *IRAK1* expression and reduced tumour size following neoadjuvant chemotherapy [117]. A synergistic effect was also noticed through the downregulated phosphatidylinositol 3,4,5-triphosphate-dependent Rac exchanger 1 (*PREX1*, FC -1.06). Briefly, decreased MCF7 viability was reported for *PREX1* loss by positive feedback to upstream phosphatidylinositol 3-kinase (P13K) activators [118]. In addition, a positive correlation was found between *PREX1* overexpression and poor prognosis in breast cancer patients via neuregulin–ErbB signalling [119]. Similarly, the neuronal guanine nucleotide exchange factor (*NGEF*) overexpression was correlated with tumour progression and metastasis, including in breast carcinomas [120]; therefore, its decreased expression in the synergistic combination-treated cells will contribute to the overall synergistic effect of CBD with SN−38 against MCF7 cells.

*PCF11*-encoded protein was among the top downregulated proteins in the combination-treated MCF7 cells, with a fold change of −4.32. *PCF11* downregulation is associated with favourable outcomes in neuroblastoma patients [121], but its functional role in breast carcinoma is still unexplored. However, it was found to be involved in the alternative polyadenylation (APA), causing three prime untranslated region (3′ UTR) shortening of mRNA by cancer-specific ubiquitin ligase [17]. Additionally, aminoacylase 1 (*ACY1*) was also significantly downregulated (FC −4.16) in the CSN38-treated MCF7 cells compared to the monotherapies. *ACY1* is responsible for amino acid deacylation during protein degradation and has been reported to be upregulated in colorectal cancer patients and HCT116 cells, promoting tumour progression [122,123]. *ACY1* has been proposed as a tumour suppressor in small-cell lung cancer, renal cell carcinoma, and hepatocellular cancer cells, as its decreased expression in these cells may accumulate acylated peptide growth factors [124,125,126]. *ACY1* was also downregulated in MCF7 cells treated with 17β-estradiol, suggesting its role as a tumour suppressor in oestrogen-dependent breast carcinomas, but further studies are needed in order to evaluate its clinical relevance in breast cancer [127].

The downregulation of integrin subunit alpha L, nucleoporin 93, and signal peptide peptidase-like 2A encoded by the *ITGAL*, *NUP93*, and *SPPL2A* genes (FC −2.01, −2.30, and −2.0, respectively) contributed to the synergistic mechanisms of CBD and SN−38 against MCF7 cells. Briefly, *ITGAL* overexpression was reported in lymph node metastases of breast cancer patients, compared to the primary breast tumours, highlighting its role in breast cancer metastasis [128]. On the other hand, the overexpression of nucleoporin 93—a nuclear envelope protein—enhanced the migration and invasion of the triple-negative and claudin-low breast cancer cells alongside metastasis in animal models [129,130]. Moreover, the signal peptide peptidase was highly induced in breast and lung cancer cell lines, and its role in tumour progression is anticipated via FKBP8 degradation [131]. Collectively, in addition to its synergistic activity against MCF7-related breast adenocarcinomas, these findings indicate the potential therapeutic role of CSN−38 against other breast cancer subtypes, including triple-negative breast cancers. However, further studies involving a wide range of in vitro and in vivo breast cancer models are necessary in order to develop potential CBD-based adjuvants for breast cancer in the future. Moreover, CBD significantly synergized the SN−38- mediated inhibition of DNA topoisomerase 1 (*TOP1*) in MCF7 cells (FC −1; *p* = 0.01 and Q = 0.03), which contributed to the overall synergy between CBD and SN−38 (Figure 8A).

## 3. Material and Methods

### 3.1. Chemicals and Drug Preparation

DOC, DOX, PTX, VIN, SN38, and CBD of > 98% purity were purchased from Sigma-Aldrich, NSW, Australia. A freshly prepared stock solution in dimethyl sulfoxide (DMSO) of 1 and 158.998 mM was prepared for chemotherapeutic drugs and CBD, respectively. CBD was combined with drugs in nine different ratios (1:9, 2:8, 3:7, 4:6, 5:5, 6:4, 7:3, 8:2, and 9:1, *v*/*v*) for combination index (CI) analyses. Moreover, 50, 1, 2, 1, and 2 mM stock in DMSO of DOC, DOX, PTX, VIN, and SN38, respectively, were prepared for synergy studies in a checkerboard design.

### 3.2. Breast Adenocarcinoma Cell Line Culture Conditions

MCF7 human breast adenocarcinoma cells were obtained from the American Type Culture Collection (ATCC: Manassas, VA, USA). Dulbecco’s modified Eagle’s medium (DMEM; Lonza, NSW, Australia) with 4.5 g/L glucose, L-glutamine, and sodium pyruvate (Lonza Australia Pty Ltd., Mount Waverley, VIC, Australia), supplemented with 10% foetal bovine serum (FBS; Interpath, Heidelberg West, VIC, Australia) and 100 U/mL of penicillin and streptomycin (Gibco^TM^ BRL, Scoresby, VIC, Australia) was used to culture MCF7 cell line at 37 °C in the presence of 5% CO_2_.

### 3.3. Cell Viability Determination

Cellular viability was determined using the alamarBlue (resazurin) assay [132,133,134]. Briefly, in a 96-well plate, 100 μL of the suspended MCF7 cells was seeded at 1 × 10^4^/well, and incubated at 37 °C in the presence of 5% CO_2_ overnight to adhere. The cells were treated with different concentrations of CBD, chemotherapeutic drugs, and their combinations in different ratios, together with the vehicle control (0.5% dimethyl sulfoxide). After 72 h, the medium was removed from the wells, and then 100 μL of alamarBlue (0.1 mg/mL in FBS free media) solution was added to each well and incubated for 4 h at 37 °C in the presence of 5% CO_2_. Using a microplate spectrophotometer (BMG CLARIO star, Victoria, Australia), the fluorescence was measured at an excitation wavelength of 555 nm and an emission wavelength of 595 nm. Cell viability was determined as a percentage of vehicle-treated cells (control; 0.5% DMSO).

### 3.4. Synergy Quantification of CBD and Standard Chemotherapeutics against MCF7 Human Breast Adenocarcinoma Cells

The potential interactions between CBD and the chemotherapeutic drugs were analysed using the combination index (CI) model and the DrugComb portal [75]. CompuSyn version 2.0 (Biosoft, San Francisco, CA, USA) was used for the CI calculations based on the median-effect equation, which was derived from the mass–action law [135,136,137]. In the current study, nine pairwise combinations of chemotherapeutic drugs with CBD were studied in a constant ratio design, with a six-point dose–response curve in 1:2 serial dilution (*n* = 3), using the CI model (Chou–Talalay method) (Table 5) and 1:10 serially diluted chemotherapeutic drugs in the checkerboard design (*n* = 3) for better exploration of the interaction over a wider range of doses. The response data obtained from the CI model were further analysed in DrugComb, where the mean percentage of cell inhibition and the concentrations of the combined drugs were used as inputs for synergy scores in different models and combination sensitivity score (CSS) evaluation.

### 3.5. Flow Cytometric Analyses of Apoptosis in MCF7 Human Breast Adenocarcinoma Cells Using Annexin V-CF Blue and 7-Aminoactinomycin D (7AAD)

The apoptotic profiles of MCF7 cells were examined using Abcam Apoptosis Detection Kit (#ab214663, Abcam, VIC, Australia) according to the manufacturer’s protocol, after 24 h of treatment with CBD, chemotherapeutic drugs, and their most synergistic combination. Briefly, MCF7 cells were seeded at a density of 1 × 10^6^ per mL in T75 cell culture flasks and treated with the vehicle control (0.5% DMSO), selected synergistic CBD+ chemotherapeutic drug combinations, and monotherapies (individual treatment with CBD or chemotherapeutic drugs). The cell culture media were collected after 24 h of treatment, and cells were detached using 0.25% *w/v* of trypsin for 3 min at 37 °C. Trypsin was neutralised with an equal volume of 10% FBS-containing DMEM, and combined with the previously collected media. Cell pellets were collected by centrifugation at 500× *g* for 5 min at room temperature (RT), rinsed twice in PBS, resuspended in 1 mL PBS, and centrifuged again for 5 min at 500× *g*. The cell pellets harvested from all treatment and control groups were instantaneously resuspended in 0.5 mL of 1X binding buffer, and 5 µL of annexin V-CF blue and 7-AAD staining solutions were added to each 100 µL of cell suspension. After 15 min of incubation in the dark, 400 µL of 1X binding buffer was added. The cells were then examined using an ACEA Biosciences NovoCyte 3000 flow cytometer (ACEA Biosciences Inc., San Diego, CA, USA). For analysis and processing, the NovoExpress (version 1.5.0, ACEA Biosciences Inc., USA) software was implemented, where cells were gated on FSC vs. SSC to exclude the cell debris and aggregates. The cells were then gated on a dot plot of Annexin V-CF in Pacific Blue vs. 7-AAD fluorescence in PerCP, with a quadrant placed indicating live cells (+Annexin V and −7-AAD) in the lower-left quadrant, early apoptotic cells (+Annexin V and −7-AAD) in the lower-right quadrant, late apoptotic cells (+Annexin V and +7-AAD) in the upper-right quadrant, and necrotic cells (−Annexin V and +7-AAD) in the upper-left quadrant. Cell percentage data from each quadrant after each treatment (*n* = 3) were exported to GraphPad Prism for statistical analysis and visualisation. Annexin V and 7-AAD (7-aminoactinomycin D) were combined to distinguish necrotic cells from early and late apoptotic cells. Annexin V binds to the phosphatidylserine (PS) phospholipids, which are translocated to the outer surface of cells during apoptosis [138]. 7-AAD, on the other hand, is a fluorescent dye that intercalates in double-stranded DNA, with a high affinity for guanine–cytosine residues, and is used as a fluorescent DNA marker in flow cytometry and fluorescence microscopy [139,140]. The PerCP and Pacific Blue channels were used for Annexin V and 7-AAD, respectively, as the emission spectra of these dyes do not overlap; therefore, no compensation is necessary [68].

### 3.6. Bottom-Up Label-Free Quantification Proteomics Study of MCF7 Cell Lysates after Treatment with the Most Synergistic Combination

#### 3.6.1. Cell Culture, Treatment, and Protein Extraction

MCF7 cells were seeded at a density of 1.0 × 10^6^ per mL cells in T75 flasks and incubated overnight at 37 °C in the presence of 5% CO_2_. The media were aspirated and replaced with fresh media containing 0.5% DMSO as the vehicle control, and the selected doses of chemotherapeutic drugs, CBD, and their synergistic combinations, in triplicate, and incubated for 24 h at 37 °C in the presence of 5% CO_2_. Each cell flask was treated with 0.25% *w/v* trypsin for 3 min at 37 °C, and the cell culture media were collected. Trypsin was neutralised with an equal volume of 10% FBS-containing DMEM before mixing with the previously collected media. The cells were centrifuged at 500× *g* for 5 min at RT, and then the pellets were rinsed twice with ice-cold PBS and centrifuged again at 500× *g* for 5 min. The cell pellets were then resuspended in 100 µL of lysis buffer including 1 µL of universal nuclease and fully mass spectrometry (MS)-compatible Halt™ Protease and Phosphatase Inhibitor Cocktail, EDTA-Free (Thermo Scientific, Waltham, MA, USA). The cells were pipetted up and down 10–15 times until the sample viscosity was reduced, and then placed on ice for 20 min. The lysate was then centrifuged at 14,000 rpm for 20 min at 4 °C and collected.

#### 3.6.2. Protein Quantification

The Pierce™ Rapid Gold BCA Protein Assay Kit (#A53226, Thermo Scientific, USA) was employed for the determination of protein concentration of the cell lysate, in triplicate, against bovine serum albumin (BSA) standard, as per the manufacturer’s protocol. Briefly, 1 µL of each sample replicate was diluted 1:20 in water together with 20 µL of each standard, and then placed in a 96-well plate with 200 µL of working reagent per well. Samples were diluted until they were within the working range of 20–2,000 µg mL^−1^. The plate was mixed thoroughly on a plate shaker for 30 s and incubated at RT for 5 min, and then the absorbance was measured within 20 min at 480 nm using a microplate spectrophotometer (BMG CLARIOstar, Melbourne, VIC, Australia). The blank absorbance was subtracted from all other readings of standards and samples, and sample concentration was determined against the established BSA standard calibration curve. Samples were stored at −80 °C for further analysis.

#### 3.6.3. Preparation and Clean-Up of Peptides

One-hundred-microgram protein samples were used for chemical and enzymatic sample processing using the EasyPep™ Mini MS Sample Prep Kit, as per the manufacturer’s protocol (Thermo Fisher Scientific, USA). The final volume was adjusted to 100 µL using lysis buffer in a microcentrifuge tube. The reduction and alkylation solutions (50 µL each) were added, gently mixed, and incubated at 95 °C using a heat block for 10 min. The samples were allowed to cool at RT, and then 50 µL of the reconstituted trypsin/lys-C protease mixture was added to each sample and incubated with shaking at 37 °C for 3 h. After incubation, 50 µL of digestion stop solution was added and mixed gently. Peptide clean-up columns were implemented to remove hydrophilic and hydrophobic contaminants. Clean peptide samples were dried using a vacuum centrifuge and resuspended in 100 µL of 0.1% formic acid in water for LC–MS analysis, and then carefully placed in maximum recovery sample vials (Waters Corp, Milford, MA, USA).

#### 3.6.4. Label-Free Bottom-Up Quantification Proteomics Analysis via Nano-Ultra-High-Performance Liquid Chromatography Coupled with Quadruple Time-of-Flight Mass Spectrometry (Nano-UPLC-qTOF-MS)

A nanoACQUITY UPLC system (Waters Corp., Milford, MA, USA) coupled with a Synapt G2-S high-definition mass spectrometer (HDMS) (Waters Corp., Manchester, UK) operating in positive electron spray ion mode (ESI+) and equipped with a hybrid quadrupole time of flight (qTOF) analyser was used to analyse tryptic peptides. A Waters NanoLockSpray Exact Mass Ionization Source was utilised to maintain mass accuracy. Briefly, 100 fg mL-1 Glu-fibrinopeptide B (GFP) dissolved in 50% aqueous acetonitrile containing 0.1% formic acid with a lock mass m/z of 785.84.26 was infused as a lock spray solution at 0.5 μL min^−1^ and calibrated against a sodium iodide solution. For the chromatographic separation of peptides, the nanoEase M/Z BEH C18 (1.7 μm, 130 Å, 75 μm × 100 mm, Waters Corp., Milford, MA, USA) at 40 °C was utilised and coupled with a nanoEase M/Z Symmetry C18 Trap Column (100 Å, 5 µm, 180 µm × 20mm, Waters Corp., Milford, MA, USA). Milli-Q water and acetonitrile containing 0.1% formic acid were used as mobile phases A and B, respectively (LCMS grade, Merck, Germany). An injection volume of 1 µL at a 300 nL min^−1^ flow rate was used throughout the 50 min gradient. Samples were injected into the trapping column at 5 μL min^−1^ at 99% mobile phase A for 3 min, before being eluted to the analytical column. An initial 1% of mobile phase B was ramped to 85% B over 50 min with the following gradient: 10% B at 2 min, 40% B at 40 min, and 85% B at 42 min. All samples were kept at 4 °C and were injected in duplicates. The ion source block temperature was set to 80 °C, and capillary voltage was maintained at 3 kV. Ions were acquired with m/z between 50 and 2000, scanning time of 0.5 sec, sample cone voltage and source offset at 30 V, nanoflow gas at 0.3 Bar, purge gas at 20 Lh^−1^, and cone gas flow at 20 L h^−1^. A data–independent acquisition (DIA) method by MS^E^ multiplex mode was used for sample acquisition with a T-wave collision-induced dissociation cell filled with argon gas, using MassLynx Mass Spectrometry Software (Waters Corporation, Milford, MA, USA).

#### 3.6.5. Data Processing and Availability

Progenesis QI software (Waters Corporation, Milford, MA, USA) was used to import and further process the MassLynx-acquired data. Automatic selection of alignment references among QC samples was set, and peptides were identified against the UniProt human proteome database (October 2020 version) using the ion accounting method, with a 250 kDa protein mass maximum. One fragment per peptide or one peptide per protein together with 3 fragments per protein were set as ion matching requirements using relative quantification, implementing the Hi-N method (*n* = 3). Auto peptide and fragment tolerance, and less than 4% FDR, were set as search tolerance parameters. Peptides with absolute mass error >20 ppm or a single charge were further filtered out. Pairwise comparisons of the identified proteins in the treated groups were performed against the control group for cytotoxic potential exploration, while the most synergistic combination samples were compared against monotherapy-treated samples for the elucidation of possible synergistic mechanisms. In each experimental design, proteins with analysis of variance (ANOVA)-derived *p*-values of at least≤ 0.05 and q-values ≤ 0.05, with a fold change ≥2, were considered significant and included for further pathway analyses. Differentially expressed proteins identified by the quantitative processing of the LC–MS/MS analysis of the proteome tryptic digestion were analysed by STRING [80], Reactome [81], g:Profiler [82], and IMPaLA [83] to identify the relevant pathways responsible for the synergistic effect against MCF7 cells. The G: SCS algorithm was used for multiple testing corrections in the g:Profiler platform, with an adjusted *p*-value threshold of 0.05. The raw and processed data have been deposited to the ProteomeXchange Consortium via the PRoteomics IDEntifications (PRIDE) repository [141], with the dataset identifiers PXD026587 and 10.6019/PXD026331.

### 3.7. Statistical Analysis

All statistical comparisons were performed using GraphPad Prism Version 9 (San Diego, CA, USA), except for the shotgun proteomics study, where MetaboAnalyst 5.0 was used together with Progenesis QIP (Waters Corporation, Milford, MA, USA). The significance was analysed via ANOVA and *t*-tests for multiple and pairwise comparisons, respectively. Data were expressed as means  ±  SD. Differences of at least *p*  <  0.05 between the mean values in the experiments were considered statistically significant.

## 4. Conclusions and Future Directions

Synergistic interactions were observed between CBD and the five selected chemotherapeutic drugs at different molar ratios in MCF7 cells, using different synergy quantitation models. We highlighted the promising molar ratios of CBD and chemotherapeutic drug combinations that were validated against different synergy metrics. CBD synergistically enhanced the antiproliferative activity of the standard chemotherapeutic drugs by promoting apoptosis. Moreover, the presented pilot label-free quantification-driven proteomics study highlighted the cytotoxic mechanisms of CBD against MCF7 cells, along with the underlying synergistic mechanisms of its combination with SN−38. However, further targeted proteomics studies with absolute quantification are warranted in order to validate the presented targets and pathways. Good tolerability and minimal adverse effects are reported for CBD in clinical trials (50–1000 mg/day up to 13 weeks), with an increased easing of legislation to approve CBD products. Nevertheless, further validation of the proposed combinations in normal, non-transformed breast epithelial cells is required.

The present study highlights potential new opportunities in breast cancer treatment with other chemotherapeutic agents, such as CDK or topoisomerase inhibitors. For example, we speculated the potential CBD combinations with CDK4/6 inhibitors such as abemaciclib, palbociclib, and ribociclib based on CBD-mediated inhibition of CDK-6 in MCF7 cells. The presented cytotoxic mechanisms of CBD are encouraging for further investigation of CBD’s interaction with hormonal therapies, including aromatase inhibitors (such as anastrozole, letrozole, and exemestane), selective oestrogen receptor modulators (SERM such as tamoxifen or toremifene), and new generations of drugs such as fulvestrant and goserelin.

Collectively, CBD presents an opportunity to potentially enhance the effectiveness of the breast cancer treatment regimens containing doxorubicin, docetaxel, paclitaxel, SN−38, and vinorelbine. Furthermore, the proposed combinations with CBD may be able to alleviate chemotherapy-induced adverse effects by reducing the dosage of chemotherapeutic drugs, or via CBD’s ameliorative or protective activities. However, further in vivo and clinical studies are warranted in order to validate these in vitro findings.

## Figures and Tables

**Table 1:** Drug interaction analysis of cannabidiol (CBD) and the chemotherapeutic combinations in MCF7 breast cancer cells.

Combo ID, Molar Ratio	CI Values at					CSS	S	ZIP	BLISS	LOEWE	HSA
	IC50	IC75	IC90	IC95	IC97						
**CBD: Docetaxel**											
CDOC19, 18:1	1.23	1.35	1.64	2.07	2.60	35.5	0.33	−11.81	−19.12	−6.84	−15.76
CDOC28, 40:1	0.96	1.14	1.62	2.32	3.18	54.6	18.07	−10.9	−13.6	**14.29**	−9.42
CDOC37, 68:1	0.70	0.91	1.46	2.27	3.27	41.33	3.96	−9.74	−11.35	−8.14	−7.08
CDOC46, 106:1	**0.49**	0.70	1.23	2.04	3.03	49.82	13.09	−3.4	−5.61	−2.36	−1.38
CDOC55, 159:1	**0.45**	0.70	1.35	2.33	3.54	52.65	14.33	−3.34	−4.58	−2.05	−0.8
CDOC64, 238:1	**0.50**	0.86	1.80	3.20	4.94	51.66	12.22	−3.04	−4.2	−1.74	−0.29
CDOC73, 371:1	**0.38**	0.72	1.61	2.95	4.61	52.44	13.36	−2.36	−3.69	−1.35	0.07
CDOC82, 636:1	**0.42**	**0.61**	1.00	1.46	1.93	**56.64**	**20.16**	**1.94**	−0.1	**1.86**	**3.4**
CDOC91, 1431:1	**0.48**	0.73	1.17	1.65	2.12	**56.04**	**20.19**	**1.96**	−1.08	0.16	**2.06**
**CI to DC**	-	-	-	-	-	**55.50**	18.89	−8.41	−7.93	−1.46	−0.65
**Checkerboard**	-	-	-	-	-	**72.91**	−11.77	1.76	−5.07	−4.17	−0.66
**Selected dose** **(39.75, 0.5 µM)**	-	-	-	-	-	-	-	**28.12**	**21.45**	**24.27**	**46.02**
**CBD: Doxorubicin**											
CDOX19, 18:1	1.17	1.16	1.15	1.14	1.14	**64.78**	**46.12**	**6.07**	**4.52**	−5.24	**1.62**
CDOX28, 40:1	1.12	1.11	1.10	1.10	1.09	**66.93**	**46.23**	**3.32**	**1.97**	−9.04	0
CDOX37, 68:1	1.07	1.06	1.05	1.05	1.05	**66.94**	**43.69**	0.18	−1.53	−10.92	−2.66
CDOX46, 106:1	1.42	1.41	1.40	1.39	1.38	**65.5**	**42.45**	0.61	−1.64	−11.27	−2.78
CDOX55, 159:1	1.34	1.33	1.32	1.31	1.31	24.4	1.7	−13.69	−46.05	−55.66	−48.18
CDOX64, 238:1	1.27	1.26	1.25	1.25	1.25	**69.85**	**45.41**	**4.15**	**2.24**	−6.37	0.99
CDOX73, 371:1	1.20	1.19	1.19	1.18	1.18	**73.33**	**48.85**	**6.67**	**5.38**	−2.87	**4.15**
CDOX82, 636:1	1.13	1.13	1.12	1.12	1.12	**75.33**	**52.37**	**10.19**	**9.06**	0.67	**7.38**
CDOX91, 1431:1	1.07	1.06	1.06	1.06	1.06	**76.85**	**55.09**	**9.89**	**8.72**	−2.69	**6.75**
**CI to DC**	-	-	-	-	-	**70.24**	**53.04**	**4.96**	**4.05**	−5.43	**2.21**
**Checkerboard**	-	-	-	-	-	54.90	−5.31	1.04	−1.05	−9.15	−4.31
**Selected dose** **(38.42, 0.2 µM)**	-	-	-	-	-	-	-	**32.52**	**25.65**	**15.16**	**31.53**
**CBD: Paclitaxel**											
CPTX19, 18:1	1.50	1.61	1.89	2.35	2.95	35.94	0.09	−12.32	−20.86	−20.59	−19.3
CPTX28, 40:1	0.91	1.04	1.40	2.00	2.78	42.63	5.16	−7.42	−12.28	−11	−9.74
CPTX37, 68:1	0.70	0.85	1.31	2.05	3.03	40.73	2.11	−8.38	−11.49	−10.07	−8.88
CPTX46, 106:1	**0.48**	**0.63**	1.09	1.83	2.82	50.48	12.66	−2.18	−5.93	−4.42	−3.42
CPTX55, 159:1	**0.37**	**0.54**	1.04	1.85	2.93	**55.54**	16.97	−0.14	−3.26	−2.93	−1.55
CPTX64, 238:1	**0.37**	**0.59**	1.24	2.32	3.75	51.98	11.68	−1.04	−4.26	−3.37	−1.96
CPTX73, 371:1	**0.34**	**0.61**	1.41	2.73	4.49	52.76	12.71	−0.76	−3.19	−2.35	−1.01
CPTX82, 636:1 *	**0.30**	**0.62**	1.57	3.14	5.24	**59.16**	**21.67**	**4.4**	**2.03**	**2.33**	**3.84**
CPTX91, 1431:1 *	**0.33**	0.80	2.21	4.56	7.68	**59.28**	**23.01**	**3.81**	1.2	1.2	**2.66**
**CI to DC**	-	-	-	-	-	**57.70**	**21.51**	−6.44	−8.30	−3.69	−3.53
**Checkerboard**	-	-	-	-	-	53.78	−31.41	−5.35	−10.85	−7.90	−6.63
**Selected dose** **(64.6, 0.1 µM)**	-	-	**0.68**	-	-	-	-	**~2.98**	~−1.69	~6.1	**~1.95**
**CBD:SN38**											
CSN19, 18:1	1.77	5.14	18.44	51.56	115.29	40.24	13.73	−0.43	−3.87	−3	−1.38
CSN28, 40:1	**0.65**	0.86	1.49	2.52	3.84	48.26	20.38	1.13	−0.58	1.23	**2.95**
CSN37, 68:1	**0.57**	0.76	1.33	2.19	3.23	45.18	15.31	−2.32	−4.31	−2.18	−0.62
CSN46, 106:1 *	**0.47**	**0.59**	0.95	1.44	1.98	50.63	21.35	1.96	0.13	**2.04**	**3.69**
CSN55, 159:1	**0.47**	**0.60**	0.94	1.37	1.82	50.13	19.75	1.08	−0.83	−0.05	**1.7**
CSN64, 238:1	**0.45**	**0.60**	0.97	1.40	1.85	49.6	18.07	0.23	−1.72	0.05	**1.64**
CSN73, 371:1 **	0.81	**0.66**	**0.61**	**0.60**	**0.59**	52.98	**21.84**	**1.83**	0.38	**1.99**	**3.57**
CSN82, 636:1 **	0.81	0.71	**0.68**	**0.67**	**0.67**	**59.38**	**29.68**	**5.05**	**3.67**	**4.45**	**6.4**
CSN91, 1431:1 **	0.92	0.93	0.91	0.91	0.90	**58.73**	**29.9**	**3.43**	**2.21**	**2.79**	**4.66**
**CI to DC**	-	-	-	-	-	48.39	**23.86**	**2.37**	**1.35**	**3.58**	**4.46**
**Checkerboard**	-	-	-	-	-	**75.94**	1.36	0.31	−3.24	−3.09	−0.08
**Selected dose** **(42.45, 0.11 µM)**	-	0.66	-	-	-	-	-	**14.55**	**23.86**	**31.38**	**33.57**
**CBD: Vinorelbine**											
CVIN19, 18:1	1.30	1.39	1.64	2.02	2.53	31.94	−1.9	−9.19	−16.9	−16.72	−15.15
CVIN28, 40:1	1.03	1.18	1.58	2.25	3.12	37.98	2.98	−8.44	−12.37	−12.2	−10.12
CVIN37, 68:1	0.71	0.86	1.30	2.02	2.96	41.71	5.92	−6.9	−8.99	−7.59	−5.91
CVIN46, 106:1	**0.56**	0.74	1.26	2.12	3.25	44.84	9.79	−4.31	−6.29	−5.35	−3.24
CVIN55, 159:1	**0.46**	**0.67**	1.27	2.26	3.58	50.93	14.65	−1.54	−3.63	−3.13	−1.06
CVIN64, 238:1	**0.45**	0.72	1.52	2.84	4.58	49.75	12.98	−2.21	−5.08	−3.64	−1.84
CVIN73, 371:1	**0.33**	**0.59**	1.36	2.63	4.32	52.5	16.13	0.16	-2.16	−1.01	1.06
CVIN82, 636:1 *	**0.32**	**0.65**	1.65	3.30	5.49	**55.57**	**21.19**	**3.31**	**1.6**	**1.94**	**4.38**
CVIN91, 1431:1 **	0.73	**0.60**	**0.56**	**0.55**	**0.55**	**56.32**	**25.34**	**5.55**	**3.36**	**2.56**	**5.09**
**CI to DC**	-	-	-	-	-	**57.45**	**23.54**	−5.89	−6.49	−1.64	−0.70
**Checkerboard**	-	-	-	-	-	**56.45**	−30.43	−1.79	−6.39	−4.94	−4.07
**Selected dose** **(42.4, 0.1 µM)**	-	**~0.62-**	-	-	-	-	-	**18.29**	**28.44**	**43.26**	**48.43**

CI = combination index; CI to DC = all combined responses from CI model combinations were analysed via DrugComb server; CSS = combination sensitivity score; IC = inhibitory concentration killing the corresponding percentage of MCF7 cells; HSA = highest single agent model; ZIP = zero interaction potency model; ** = synergistic combinations in all models and inhibitory concentrations; * = synergistic combinations in all models and most effective doses; ~ = approximate score considered. Potential synergistic combinations with CI < 0.7 or synergy score > 1.5 in Loewe, HSA, ZIP, and Bliss models, or > 20 in the S model, are bold formatted. Potential effective combinations (CSS > 55) are bold formatted. Synergy metrics listed for the selected doses are derived from CI to DC.

**Table 2:** Significantly upregulated proteins in cannabidiol-treated MCF7 cells.

Uniprot ID *	HGNC Gene ID	Protein Name	Log2 Fold Change
P04733	*MT1F*	Metallothionein-1F	7.03
A0A0J9YWD4	*RIMS2*	Regulating synaptic membrane exocytosis protein 2	5.80
Q8WX94	*NLRP7*	NACHT_ LRR and PYD domains-containing protein 7	5.75
P02795	*MT2A*	Metallothionein-2	3.78
Q9Y2Z9	*COQ6*	Ubiquinone biosynthesis monooxygenase COQ6	2.60
A6NCE7	*MAP1LC3B2*	Microtubule-associated proteins 1A/1B light chain 3 beta 2	1.68
E9PRY0	*EIF3M*	Eukaryotic translation initiation factor 3 subunit M	1.66
Q9NX24	*NHP2*	H/ACA ribonucleoprotein complex subunit 2	1.51
Q5JPF3	*ANKRD36C*	Ankyrin repeat domain-containing protein 36C	1.50
A0A0B4J1X2	*CACNA1G*	Voltage-dependent T-type calcium channel subunit α-1G	1.48
P12645	*BMP3*	Bone morphogenetic protein 3	1.45
Q9BQ39	*DDX50*	ATP-dependent RNA helicase DDX50	1.44
P08910	*ABHD2*	Monoacylglycerol lipase ABHD2	1.40
Q8TCU6	*PREX1*	Phosphatidylinositol 3_4_5-trisphosphate-dependent Rac exchanger 1 protein	1.35
Q3KQV9	*UAP1L1*	UDP-N-acetylhexosamine pyrophosphorylase-like protein 1	1.34
Q14139	*UBE4A*	Ubiquitin conjugation factor E4 A	1.26
M0QZQ3	*SPTBN4*	Spectrin beta chain	1.26
F5GZS6	*SLC3A2*	4F2 cell-surface antigen heavy chain	1.25
P35527	*KRT9*	Keratin_ type I cytoskeletal 9	1.23
F5GX23	*PSMD9*	26S proteasome non-ATPase regulatory subunit 9	1.22
Q5XKE5	*KRT79*	Keratin_ type II cytoskeletal 79	1.07
Q9NRV9	*HEBP1*	Heme-binding protein 1	1.05
H0YMI4	*USP3*	Ubiquitin carboxyl-terminal hydrolase	1.01
B4DIG0	*CDC25B*	Protein-tyrosine-phosphatase	1.00
P29992	*GNA11*	Guanine nucleotide-binding protein subunit alpha-11	1.00

* Significantly upregulated proteins in 42.45 µM CBD-treated MCF7 cells (*p* and Q ≤ 0.01 alongside Progenesis QIP-calculated maximum fold change ≥ 2 = log2 fold change ≥ 1) compared to the vehicle-treated control cells.

**Table 3:** Significantly downregulated proteins in cannabidiol-treated MCF7 cells.

Uniprot ID *	HGNC Gene ID	Protein Name	Log2 Fold Change
P02538	*KRT6A*	Keratin 6A	−28.33
E9PC85	*LRRK2*	Leucine-rich repeat kinase 2	−30.08
Q9BRK5	*SDF4*	45 kDa calcium-binding protein	−10.86
P28331	*NDUFS1*	NADH-ubiquinone oxidoreductase 75 kDa subunit_ mitochondrial	−8.44
K7EPA3	*TMEM161A*	Transmembrane protein 161A	−7.83
Q14249	*ENDOG*	Endonuclease G_ mitochondrial	−7.53
Q9H501	*ESF1*	ESF1 homologue	−4.39
Q9NRY5	*FAM114A2*	Protein FAM114A2	−4.24
C9J2C7	*NT5C1B-RDH14*	NT5C1B-RDH14 readthrough	−3.95
Q9NX14	*NDUFB11*	NADH dehydrogenase [ubiquinone] 1 beta subcomplex subunit 11_ mitochondrial	−3.49
A0A3B3ISF0	*LARP1B*	La-related protein 1B	−3.35
Q9UP79	*ADAMTS8*	A disintegrin and metalloproteinase with thrombospondin motifs 8	−3.34
Q8IYA6	*CKAP2L*	Cytoskeleton-associated protein 2-like	−3.31
P60602	*ROMO1*	Reactive oxygen species modulator 1	−3.26
P55317	*FOXA1*	Hepatocyte nuclear factor 3-alpha	−3.25
Q96G01	*BICD1*	Protein bicaudal D homologue 1	−3.23
O43291	*SPINT2*	Kunitz-type protease inhibitor 2	−3.21
O00762	*UBE2C*	Ubiquitin-conjugating enzyme E2 C	−3.04
O00291	*HIP1*	Huntingtin-interacting protein 1	−2.83
Q02818	*NUCB1*	Nucleobindin-1	−2.81
E9PJC6	*AHNAK*	Neuroblast differentiation-associated protein AHNAK	−2.58
Q2TV78	*MST1L*	Putative macrophage stimulating 1-like protein	−2.51
A0A6Q8PFY3	*FANCD2*	Fanconi anemia group D2 protein	−2.45
A0A0U1RR07	*SYTL2*	Synaptotagmin-like protein 2	−2.43
Q8IVV2	*LOXHD1*	Lipoxygenase homologuey domain-containing protein 1	−2.31
Q9BUB7	*TMEM70*	Transmembrane protein 70_ mitochondrial	−2.28
Q8TCT8	*SPPL2A*	Signal peptide peptidase-like 2A	−2.27
Q96DV4	*MRPL38*	39S ribosomal protein L38_ mitochondrial	−2.27
Q9Y646	*CPQ*	Carboxypeptidase Q	−2.23
Q15036	*SNX17*	Sorting nexin-17	−2.22
O75306	*NDUFS2*	NADH dehydrogenase [ubiquinone] iron–sulfur protein 2_ mitochondrial	−2.17
G3V0I5	*NDUFV1*	NADH dehydrogenase [ubiquinone] flavoprotein 1_ mitochondrial	−2.12
A0A590UJ21	*CUL1*	Cullin-1	−1.97
Q00534	*CDK6*	Cyclin-dependent kinase 6	−1.96
Q9P1A6	*DLGAP2*	Disks large-associated protein 2	−1.95
K7EJV0	*SEPTIN9*	Septin-9	−1.92
E7EN73	*KIAA0319L*	Dyslexia-associated protein KIAA0319-like protein	−1.92
C9JJ19	*MRPS34*	28S ribosomal protein S34_ mitochondrial	−1.92
Q92665	*MRPS31*	28S ribosomal protein S31_ mitochondrial	−1.88
Q96C01	*FAM136A*	Protein FAM136A	−1.86
Q16540	*MRPL23*	39S ribosomal protein L23_ mitochondrial	−1.83
A0A2R8YGD3	*RAPGEF2*	Cyclic nucleotide ras GEF	−1.81
G8JLA1	*RDH13*	Retinol dehydrogenase 13	−1.80
P82650	*MRPS22*	28S ribosomal protein S22_ mitochondrial	−1.76
O00217	*NDUFS8*	NADH dehydrogenase [ubiquinone] iron–sulfur protein 8_ mitochondrial	−1.75
Q9Y2Z2	*MTO1*	Protein MTO1 homologue_ mitochondrial	−1.68
A0A0A0MRM2	*NRAP*	Nebulin-related-anchoring protein	−1.64
Q9UKX2	*MYH2*	Myosin-2	−1.64
Q6X4W1	*NSMF*	NMDA receptor synaptonuclear signaling and neuronal migration factor	−1.62
O75363	*BCAS1*	Breast carcinoma-amplified sequence 1	−1.62
O94913	*PCF11*	Pre-mRNA cleavage complex 2 protein Pcf11	−1.62
Q92823	*NRCAM*	Neuronal cell adhesion molecule	−1.60
Q96EY7	*PTCD3*	Pentatricopeptide repeat domain-containing protein 3_ mitochondrial	−1.57
Q6ZXV5	*TMTC3*	Protein O-mannosyl-transferase TMTC3	−1.53
Q6IBS0	*TWF2*	Twinfilin-2	−1.50
Q9NY74	*ETAA1*	Ewing’s tumour-associated antigen 1	−1.50
Q8N961	*ABTB2*	Ankyrin repeat and BTB/POZ domain-containing protein 2	−1.42
A0A1B0GU86	*ACY1*	N-acyl-L-amino-acid amidohydrolase	−1.42
P55011	*SLC12A2*	Solute carrier family 12 member 2	−1.42
H7BYU6	*ZNF521*	Zinc finger protein 521	−1.38
P04181	*OAT*	Ornithine aminotransferase_ mitochondrial	−1.36
Q8N565	*MREG*	Melanoregulin	−1.33
Q14802	*FXYD3*	FXYD domain-containing ion transport regulator 3	−1.33
Q7L2E3	*DHX30*	ATP-dependent RNA helicase DHX30	−1.32
Q12767	*TMEM94*	Transmembrane protein 94	−1.31
Q8N0X2	*SPAG16*	Sperm-associated antigen 16 protein	−1.30
P14854	*COX6B1*	Cytochrome c oxidase subunit 6B1	−1.30
Q92820	*GGH*	Gamma-glutamyl hydrolase	−1.28
M0QY24	*ZNF546*	Zinc finger protein 546	−1.26
Q02880	*TOP2B*	DNA topoisomerase 2-beta	−1.25
P50579	*METAP2*	Methionine aminopeptidase 2	−1.23
Q8NEZ3	*WDR19*	WD repeat-containing protein 19	−1.23
G5E9Z9	*LRP2BP*	LRP2 binding protein_ isoform CRA_a	−1.22
Q8TDY2	*RB1CC1*	RB1-inducible coiled-coil protein 1	−1.22
H0Y5K5	*ERGIC3*	Endoplasmic reticulum-Golgi intermediate compartment protein 3	−1.20
Q9H869	*YY1AP1*	YY1-associated protein 1	−1.20
A7XYQ1	*SOBP*	Sine oculis-binding protein homologue	−1.18
P49006	*MARCKSL1*	MARCKS-related protein	−1.18
H3BLV9	*SRPK1*	SRSF protein kinase 1	−1.17
Q9Y487	*ATP6V0A2*	V-type proton ATPase 116 kDa subunit a2	−1.16
Q9BZG8	*DPH1*	2-(3-amino-3-carboxypropyl)histidine synthase subunit 1	−1.15
H3BV16	*NDUFB10*	Complex I-PDSW	−1.15
Q99797	*MIPEP*	Mitochondrial intermediate peptidase	−1.15
O95294	*RASAL1*	RasGAP-activating-like protein 1	−1.14
P23921	*RRM1*	Ribonucleoside-diphosphate reductase large subunit	−1.14
Q8IVG5	*SAMD9L*	Sterile alpha motif domain-containing protein 9-like	−1.13
P31350	*RRM2*	Ribonucleoside-diphosphate reductase subunit M2	−1.13
Q8WVV9	*HNRNPLL*	Heterogeneous nuclear ribonucleoprotein L-like	−1.08
A0A2R8YET2	*PRRC2C*	Protein PRRC2C	−1.07
Q9HCM1	*RESF1*	Retroelement silencing factor 1	−1.07
F8VWW7	*SPRYD3*	SPRY domain-containing protein 3	−1.06
P54296	*MYOM2*	Myomesin-2	−1.03
Q86XE3	*MICU3*	Calcium uptake protein 3_ mitochondrial	−1.02
P49736	*MCM2*	DNA replication licensing factor MCM2	−1.01
Q9H4E5	*RHOJ*	Rho-related GTP-binding protein RhoJ	−1.01
Q9HAZ2	*PRDM16*	Histone-lysine N-methyltransferase PRDM16	−1.00

* Significantly downregulated proteins in 42.45µM CBD-treated MCF7 cells (*p* and Q ≤ 0.01 alongside maximum Progenesis QIP-calculated maximum fold change ≥2 = log2 fold change ≤−1) compared to the vehicle-treated control cells.

**Table 4:** Significantly downregulated proteins in CSN38-treated MCF7 cells compared to the monotherapies.

UniProt ID	HGNC Gene ID	Description	Log2 Fold Change *
**Downregulated proteins**			
O75363	*BCAS1*	Breast carcinoma-amplified sequence 1	−27.75
Q9NVH1	*DNAJC11*	DnaJ homologue subfamily C member 11	−24.68
O00762	*UBE2C*	Ubiquitin-conjugating enzyme E2 C	−4.84
O94913	*PCF11*	Pre-mRNA cleavage complex 2 protein Pcf11	−4.32
A0A1B0GU86	*ACY1*	N-acyl-L-amino-acid amidohydrolase	−4.16
A0A2R8YGD3	*RAPGEF2*	Cyclic nucleotide ras GEF	−2.48
Q9P1V8	*SAMD15*	Sterile alpha motif domain-containing protein 15	−2.44
Q8TDI0	*CHD5*	Chromodomain-helicase-DNA-binding protein 5	−2.42
K7ER88	*ACAA2*	3-Ketoacyl-CoA thiolase_ mitochondrial (Fragment)	−2.05
P20701	*ITGAL*	Integrin alpha-L	−2.01
A0A0D9SG95	*CCT7*	T-complex protein 1 subunit eta	−1.78
P03952	*KLKB1*	Plasma kallikrein	−1.72
Q92823	*NRCAM*	Neuronal cell adhesion molecule	−1.63
O75582	*RPS6KA5*	Ribosomal protein S6 kinase alpha-5	−1.56
Q6UXG2	*ELAPOR1*	Endosome/lysosome-associated apoptosis and autophagy regulator 1	−1.56
Q5H9M0	*PWWP3B*	PWWP domain-containing DNA repair factor 3B	−1.47
P55199	*ELL*	RNA polymerase II elongation factor ELL	−1.46
Q9NPB8	*GPCPD1*	Glycerophosphocholine phosphodiesterase GPCPD1	−1.46
Q2TV78	*MST1L*	Putative macrophage stimulating 1-like protein	−1.42
A0A0U1RQX8	*CBL*	E3 ubiquitin-protein ligase CBL	−1.35
Q6P4H8	*ATPSCKMT*	ATP synthase subunit C lysine N-methyltransferase	−1.34
O14513	*NCKAP5*	Nck-associated protein 5	−1.33
Q8WVV9	*HNRNPLL*	Heterogeneous nuclear ribonucleoprotein L-like	−1.32
A0A2R8Y5P9	*SHROOM3*	Protein Shroom3	−1.22
O43303	*CCP110*	Centriolar coiled-coil protein of 110 kDa	−1.18
Q9C091	*GREB1L*	GREB1-like protein	−1.13
Q9BQ52	*ELAC2*	Zinc phosphodiesterase ELAC protein 2	−1.12
A0A075B757	*NBPF14*	Neuroblastoma breakpoint family member 14	−1.11
O14497	*ARID1A*	AT-rich interactive domain-containing protein 1A	−1.10
Q96C90	*PPP1R14B*	Protein phosphatase 1 regulatory subunit 14B	−1.04
Q6IEG0	*SNRNP48*	U11/U12 small nuclear ribonucleoprotein 48 kDa protein	−1.00
**Upregulated proteins**			
O94973	*AP2A2*	AP-2 complex subunit alpha-2	1.20
O14949	*UQCRQ*	Cytochrome b-c1 complex subunit 8	1.11
A0A1C7CYZ1	*MAPK15*	Mitogen-activated protein kinase 15 (Fragment)	1.10
Q5T9A4	*ATAD3B*	ATPase family AAA domain-containing protein 3B	1.03
P49189	*ALDH9A1*	4-Trimethylaminobutyraldehyde dehydrogenase	1.01

*: *p* and Q *≤* 0.01 alongside maximum Progenesis QIP-calculated fold change ≥ 2 (absolute log2 fold change ≥ 1), compared to the monotherapies (CBD and SN−38); CSN38: the synergistic combination of 42.45 µM cannabidiol and 0.11 µM SN−38.

**Table 5:** Molar ratios, concentrations, and codes of cannabidiol (CBD) combinations.

Combination Code *	Highest Concentration (µM)		Molar Ratio (CBD:Drug)
	CBD	Drug	
CXYZ19	15.9	0.9	18:1
CXYZ28	31.8	0.8	40:1
CXYZ37	47.7	0.7	68:1
CXYZ46	63.6	0.6	106:1
CXYZ55	79.5	0.5	159:1
CXYZ64	95.4	0.4	238:1
CXYZ73	111.3	0.3	371:1
CXYZ82	127.2	0.2	636:1
CXYZ91	143.1	0.1	1431:1

*: C refers to CBD; XYZ indicates initials of the chemotherapeutic drugs; the two-digit numbers refer to the combination with the corresponding molar ratio.

## Data Availability

Shotgun proteomics data are available in the PRIDE repository with the dataset identifier PXD026587, and DOI:10.6019/PXD026587. All other data are presented within the article, or in Supplementary Files 1 and 2.

## Supplementary Materials

The following are available online at https://www.mdpi.com/article/10.3390/ijms221810103/s1.

## Abbreviations

4. EBP1	Eukaryotic translation initiation factor 4E-binding protein 1
5-HT1A	Serotonin 1A receptor
AKT	Protein kinase B
CBD	Cannabidiol
CB1 and CB2	Cannabinoid receptor 1 and 2
CCL3	Chemokine (C-C motif) ligand 3
CDK	Cyclin-dependent kinase
CSS	Combination sensitivity score
DOC	Docetaxel
DOX	Doxorubicin
EGFR	Epidermal growth factor receptor
ER+/−	Oestrogen receptor-positive/negative
ETC	Electron transport chain
FC	Fold change
GM-CSF	Granulocyte-macrophage colony-stimulating factor
GPR55	G protein-coupled receptor 55
Id1	DNA-binding protein inhibitor ID-1
HER2+	Human epidermal growth factor receptor 2 positive
HER3	Human epidermal growth factor receptor 3
HGNC	HUGO Gene Nomenclature Committee
MCM	Minichromosome maintenance proteins
MIP-2	Macrophage Inflammatory Protein-2
mTOR	Mechanistic target of rapamycin
NF-κB	Nuclear factor kappa-light-chain-enhancer of activated B cells
ROS	Reactive oxygen species
PPAR-γ	Peroxisome proliferator-activated receptor gamma
PTX	Paclitaxel
SN−38	7-Ethyl-10-hydroxycamptothecin
TAM	Tumour-associated macrophage
TRPVs	Family of transient receptor potential (TRP) ion channel
TNBC	Triple-negative breast cancer
VIN	Vinorelbine
