# Supplementary material for: Synergistic Interactions of Cannabidiol with Chemotherapeutic Drugs in MCF7 Cells: Mode of Interaction and Proteomics Analysis of Mechanisms
Source: Int J Mol Sci. 2021 Sep 18;22(18):10103. doi: 10.3390/ijms221810103 (PMC8469885; doi:10.3390/ijms221810103)
Supplement: Supplementary file 1 [file ijms-22-10103-s001.zip › Supplementary file 1.pdf]

Synergistic interaction of cannabidiol with chemotherapeutic drugs in MCF7 cells: mode of interaction and proteomics analysis of mechanisms

Muhammad A. Alsherbiny [1,2,*], Deep J. Bhuyan [1,*], Mitchell N. Low[1], Dennis Chang [1] and Chun Guang Li [1,*]

[1] NICM Health Research Institute, Western Sydney University, Penrith NSW 2747, Australia; D.Chang@westernsydney.edu.au
[2] Department of Pharmacognosy, Faculty of Pharmacy, Cairo University, Cairo 11562, Egypt
* Correspondence: Muhammad.alsherbiny@pharma.cu.edu.eg (M.A.A); D.Bhuyan@westernsydney.edu.au/ deepjyoti.bhuyan@uon.edu.au (D.J.B.); c.li@westernsydney.edu.au (C.G.L.)

Table of Contents

Table S1 IC50 of cannabidiol chemotherapeutic combinations against MCF7 2

Table S2 Pearson and Spearman correlation coefficients and corresponding P values among different synergy metrics 4

Table S3 Percentage of necrotic, apoptotic, and living MCF7 cells retrieved from Annexing V-7AAD flowcytometry apoptosis analysis among CBD chemotherapeutic combinations and mono treatments 6

Figure S1 MCF7 cell percentage analysis in different treatment groups in triplicates. 7

Table S1 IC50 of cannabidiol chemotherapeutic combinations against MCF7

	GraphPad		Compusyn		
	IC50 (μgml-1)	R^2	Dm(IC50 (μgml-1))	m	r
CBD	11.75±2.82	0.96±0.03	20.48	8.01	0.93
CDOC19	5.34±1.32	0.92±0.09	0.17	1.01	1.00
CDOC28	1.74±2.46	0.08±0.07	1.55	1.00	1.00
CDOC37	~3.18±2.33	0.22±0.19	2.36	1.00	1.00
CDOC46	~2.74±1.31	0.45±0.09	2.67	1.00	1.00
CDOC55	~3.68±0.8	0.49±0.12	2.61	1.00	1.00
CDOC64	~4.59±1.43	0.62±0.07	3.15	0.99	1.00
CDOC73	6.86±3.18	0.71±0.11	4.48	0.99	1.00
CDOC82	8.25±2.98	0.82±0.07	4.27	0.99	1.00
CDOC91	11.74±3.09	0.91±0.06	5.73	1.48	0.93
DOC	0.0011±0.0005	0.84±0.18	8.12 ?	1.65	0.93
CBD	10.99±2.46	0.98±0	33.08	85.57	0.88
CDOX19	2.48±0.55	0.93±0.04	0.22	47.63	0.67
CDOX28	5.03±0.89	0.93±0.04	2.68	75.41	0.83
CDOX37	6.78±1.72	0.98±0.02	5.08	75.52	0.83
CDOX46	9.01±2.92	0.97±0.01	7.47	75.77	0.84
CDOX55	7.87±1.53	0.99±0.01	13.70	85.08	0.88
CDOX64	8.51±2.45	0.98±0.02	16.87	85.38	0.88
CDOX73	8.49±2.78	0.99±0.02	20.11	85.44	0.88
CDOX82	9.16±2.91	0.99±0.01	23.31	85.54	0.88
CDOX91	10.87±3.6	0.99±0.01	26.55	85.58	0.88
DOX	0.24±0.07	0.9±0.02	29.85 ?	85.56	0.88
CBD	10.61±1.28	0.97±0.01	33.03	85.61	0.88
CPTX19	8.19±4.06	0.67±0.18	0.16	1.01	1.00
CPTX28	~2.53±2.04	0.19±0.01	1.76	1.00	1.00
CPTX37	~3.41±2.2	0.32±0.05	2.14	1.00	1.00
CPTX46	~3.73±1.96	0.56±0.04	2.60	1.00	1.00
CPTX55	4.44±1.61	0.7±0.06	2.58	1.00	1.00
CPTX64	6.72±2	0.78±0.04	2.80	1.00	1.00
CPTX73	7.38±1.58	0.87±0.08	3.68	0.99	1.00
CPTX82	7.9±1.77	0.94±0.03	4.48	0.99	1.00
CPTX91	8.88±1.47	0.95±0.04	5.30	0.99	1.00
PTX	0.0018±0.001	0.67±0.1	7.84 ?	0.99	1.00
CBD	10.65±5.45	0.99±0	23.73	76.23	0.84
CSN19	1.12±0.56	0.77±0.06	0.19	1.01	1.00
CSN28	3.84±0.8	0.78±0.13	4.55	0.56	0.92
CSN37	5.94±1.02	0.8±0.14	3.19	1.05	0.87
CSN46	6.2±1.07	0.9±0.06	4.19	1.20	0.88
CSN55	7.72±1.57	0.94±0.02	4.57	1.48	0.94
CSN64	8.47±1.96	0.95±0.02	5.65	1.69	0.95

CSN73	8.6±2.26	0.97±0	6.42	1.78	0.96
CSN82	8.99±2.84	0.97±0.01	13.56	48.32	0.67
CSN91	10.88±3.42	0.99±0.01	15.47	48.45	0.67
SN-38	0.04±0.02	0.94±0.04	21.40	76.28	0.84
CBD	13.84±6.94	0.98±0.02	33.29	85.41	0.88
CVIN19	~5.17	0.3	0.15	1.01	1.00
CVIN28	~0.48±0.62	0.2±0.11	1.49	1.00	1.00
CVIN37	~2.68±0.83	0.45±0.13	2.38	1.00	1.00
CVIN46	¬3.44±0.76	0.58±0.02	2.63	1.01	1.00
CVIN55	~4.51±0.61	0.65±0.05	2.98	1.00	1.00
CVIN64	~7.19±3.22	0.68±0.15	3.42	0.99	1.00
CVIN73	7.38±3.19	0.76±0.09	4.50	0.99	1.00
CVIN82	9.17±3.61	0.84±0.08	4.34	0.99	1.00
CVIN91	10.95±2.9	0.94±0.01	5.59	0.99	1.00
VIN	0.0096±0.0033	0.82±0.08	17.40 ?	48.24	0.67

IC50= Concentration inhibiting 50% of MCF7 cells, R^2 or r = linear correlation coefficient representing goodness of fitting (where 1 is a perfect fit), m=kinetic order reflecting the shape of the fit curve in median effect equation, Dm= the Compusyn calculated IC_{50}

Table S2 Pearson and Spearman correlation coefficients and corresponding P values among different synergy metrics

Pearson correlation

Correlation coefficient r

	CI.at.IC50	CI.at.IC75	CI.at.IC97	CI.at.IC90	CI.at.IC95	ZIP	CSS	S	LOEWE	BLISS	HSA
CI.at.IC50	1										
CI.at.IC75	0.71	1									
CI.at.IC97	0.34	0.9	1								
CI.at.IC90	0.39	0.93	1	1							
CI.at.IC95	0.34	0.9	1	1	1						
ZIP	-0.15	-0.12	-0.0029	-0.041	-0.014	1					
CSS	-0.034	-0.18	-0.19	-0.2	-0.19	0.86	1				
S	0.23	-0.0062	-0.11	-0.11	-0.11	0.83	0.93	1			
LOEWE	-0.45	-0.2	0.036	-0.0011	0.032	0.52	0.5	0.29	1		
BLISS	-0.26	-0.16	-0.00097	-0.039	-0.0099	0.9	0.83	0.71	0.77	1	
HSA	-0.39	-0.2	0.017	-0.027	0.0087	0.82	0.72	0.56	0.86	0.98	1

***P* values (Pearson)**

	CI.at.IC50	CI.at.IC75	CI.at.IC97	CI.at.IC90	CI.at.IC95	ZIP	CSS	S	LOEWE	BLISS	HSA
CI.at.IC50	0										
CI.at.IC75	5.9e-08	0									
CI.at.IC97	0.021	3.7e-17	0								
CI.at.IC90	0.0078	8.7e-20	7.5e-49	0							
CI.at.IC95	0.021	2.6e-17	7.9e-72	9.8e-53	0						
ZIP	0.33	0.45	0.98	0.79	0.93	0					
CSS	0.82	0.23	0.22	0.18	0.2	6e-14	0				
S	0.12	0.97	0.49	0.46	0.45	2.4e-12	1.4e-20	0			
LOEWE	0.002	0.2	0.81	0.99	0.83	0.00027	0.00047	0.058	0		
BLISS	0.08	0.3	0.99	0.8	0.95	5.2e-17	2.3e-12	4.2e-08	5.3e-10	0	
HSA	0.0085	0.19	0.91	0.86	0.95	8.4e-12	2.3e-08	5.7e-05	2.2e-14	3.2e-30	0

Spearman correlation

Correlation coefficient r

	CSS	S	ZIP	BLISS	LOEWE	HSA	CI.at.IC50	CI.at.IC75	CI.at.IC90	CI.at.IC95	CI.at.IC97
CSS	1										
S	0.9	1									
ZIP	0.83	0.91	1								
BLISS	0.85	0.93	0.99	1							
LOEWE	0.42	0.5	0.55	0.56	1						
HSA	0.69	0.8	0.9	0.92	0.78	1					
CI.at.IC50	-0.13	0.073	0.081	0.05	-0.27	-0.065	1				
CI.at.IC75	-0.16	0.021	0.014	-0.0047	-0.25	-0.092	0.93	1			
CI.at.IC90	-0.49	-0.4	-0.31	-0.32	0.076	-0.17	0.39	0.56	1		

CI.at.IC95	-0.41	-0.36	-0.19	-0.19	0.34	0.047	0.081	0.21	0.86	1	
CI.at.IC97	-0.35	-0.3	-0.11	-0.12	0.36	0.12	0.061	0.17	0.8	0.98	1
***P* values (Spearman)**											
	CSS	S	ZIP	BLISS	LOEWE	HSA	CI.at.IC50	CI.at.IC75	CI.at.IC90	CI.at.IC95	CI.at.IC97
CSS	0										
S	2.4e-17	0									
ZIP	1.4e-12	2.1e-18	0								
BLISS	1.7e-13	6.6e-20	2.9e-35	0							
LOEWE	0.0036	0.00052	0.00011	5.3e-05	0						
HSA	1.2e-07	5.6e-11	3.7e-17	4e-19	3e-10	0					
CI.at.IC50	0.4	0.63	0.6	0.74	0.071	0.67	0				
CI.at.IC75	0.29	0.89	0.93	0.98	0.096	0.55	2.1e-20	0			
CI.at.IC90	0.00064	0.006	0.037	0.035	0.62	0.27	0.0085	7e-05	0		
CI.at.IC95	0.0052	0.016	0.21	0.21	0.024	0.76	0.6	0.16	2.6e-14	0	
CI.at.IC97	0.017	0.049	0.46	0.44	0.014	0.45	0.69	0.25	3.2e-11	3.2e-34	0

Table S3 Percentage of necrotic, apoptotic, and living MCF7 cells retrieved from Annexing V-7AAD flowcytometry apoptosis analysis among CBD chemotherapeutic combinations and mono treatments

**CBD combination with Docetaxel**				
	**Control**	**CBD for CDOC**	**DOC**	**CDOC**
% live cells	92.66±0.17 [a]	75.52±1.27 [b]	71.64±0.6 [c]	51.39±0.34 [d]
% Early apoptotic cells	1.12±0.11 [a]	4.36±0.24 [b]	7.11±0.9 [c]	19.62±1.43 [d]
% Late apoptotic	4.81±0.26 [a]	15.78±1.45 [b]	16.16±0.72 [b]	20.51±1.02 [c]
% Necrotic cells	1.4±0.32 [a]	4.34±0.08 [b]	5.1±0.36 [b]	8.47±0.22 [c]
% Total popototic cells	5.94±0.17 [a]	20.14±1.34 [b]	23.27±0.26 [c]	40.14±0.56 [d]
**CBD combination with Doxorubicin**				
	**Control**	**CBD for CDOX**	**DOX**	**CDOX**
% live cells	92.66±0.17 [a]	73.67±5.64 [b]	42.92±0.74 [c]	8.96±0.87 [d]
% Early apoptotic cells	1.12±0.11 [a]	8.03±1.86 [a]	8.93±2.18 [a]	20.36±6.51 [b]
% Late apoptotic	4.81±0.26 [a]	16.62±3.21 [b]	18.08±7.68 [c]	29.01±4.11 [d]
% Necrotic cells	1.4±0.32 [a]	1.69±0.57 [a]	30.06±9.47 [b]	41.68±10.19 [c]
% total apoptotic cells	5.94±0.17 [a]	24.65±5.07 [b]	27.02±9.79 [b]	49.37±10.62 [c]
**CBD combination with Paclitaxel**				
	**Control**	**CBD for CPTX**	**PTX**	**CPTX**
% live cells	92.66±0.17 [a]	32.37±1.12 [b]	89.86±0.5 [c]	23.35±0.66 [d]
% Early apoptotic cells	1.12±0.11 [a]	22.77±1.46 [b]	3.5±0.07 [c]	26.69±0.89 [d]
% Late apoptotic	4.81±0.26 [a]	36.17±0.95 [b]	5.84±0.44 [a]	38.84±1.16 [c]
% Necrotic cells	1.4±0.32 [a]	8.69±0.08 [b]	0.8±0.07 [a]	11.11±0.33 [c]
% Total apoptotic cells	5.94±0.17 [a]	58.94±1.1 [b]	9.34±0.51 [c]	65.53±0.52 [d]
**CBD combination with SN-38**				
	**Control**	**CBD for CSN-38**	**SN-38**	**CSN-38**
% live cells	92.66±0.17 [a]	78.74±0.71 [b]	89.93±0.76 [c]	60.9±3.57 [d]
% Early apoptotic cells	1.12±0.11 [a]	3.46±0.05 [b]	4.96±0.53 [a]	12.92±1.92 [c]
% Late apoptotic	4.81±0.26 [a]	11.93±0.32 [b]	3.94±0.24 [a]	19.12±1.42 [c]
% Necrotic cells	1.4±0.32 [a]	5.88±0.38 [b]	1.16±0.07 [a]	7.06±0.63 [b]
% Total apoptotic cells	5.94±0.17 [a]	15.38±0.36 [b]	8.9±0.77 [a]	32.04±3.21 [c]
**CBD combination with Vinorelbine**				
	**Control**	**CBD for CVIN**	**VIN**	**CVIN**
% live cells	92.66±0.17 [a]	71.31±0.6 [b]	62.98±0.59 [c]	55.47±0.73 [d]
% Early apoptotic cells	1.12±0.11 [b]	7.49±0.36 [b]	8.32±0.25 [b]	30.14±0.73 [c]
% Late apoptotic	4.81±0.26 [a]	15.14±0.31 [b]	16.61±0.15 [c]	10.83±0.54 [d]
% Necrotic cells	1.4±0.32 [a]	6.05±0.56 [b]	12.1±0.64 [c]	3.56±0.12 [d]
% Total apoptotic cells	5.94±0.17 [a]	22.63±0.45 [b]	24.93±0.13 [c]	40.96±0.72 [d]

Superscript letters indicate statistical significance derived from two-way ANOVA and Tukey's multiple comparisons where different letters within the same row are statistically significant with $P < 0.05$, n=3.

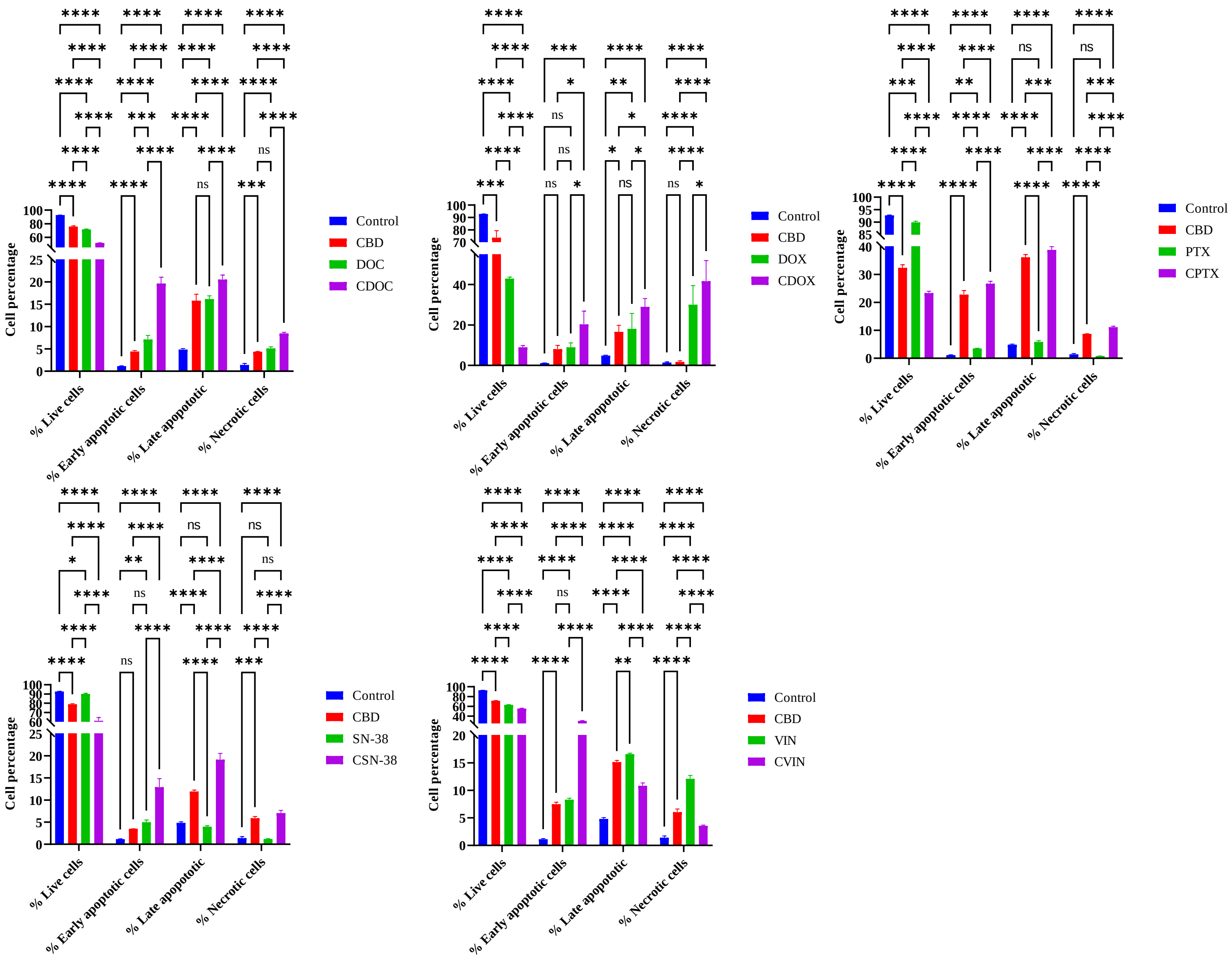

Figure S1 MCF7 cell percentage analysis in different treatment groups in triplicates.

Twenty-four hours of treatment of CBD chemotherapeutic combinations with a negative control were implemented using antibodies against Annexin-V CF-Blue and the reporter 7AAD. ****; significantly different as derived from Two-way ANOVA and Tukey's multiple comparisons at $P < 0.0001$, ***; $P < 0.001$, **; $P < 0.01$, *; $P < 0.05$, ns; non-significant.
